# Catchment Productivity Controls Local Species Richness of Hyporheic Invertebrate Communities in Tropical New Caledonia Streams

**DOI:** 10.1002/ece3.72798

**Published:** 2025-12-23

**Authors:** Samuel Mouron, Yannick Dominique, David Eme, Nina Tombers, Diana M. P. Galassi, Pierre Marmonier, Michel Lafont, Colin Issartel, Christophe J. Douady, Florian Malard

**Affiliations:** ^1^ Université Claude Bernard Lyon 1, LEHNA UMR 5023, CNRS, ENTPE Villeurbanne France; ^2^ Bio eKo Consultants Nouméa New Caledonia; ^3^ INRAE UR‐RiverLY, Centre Lyon‐Grenoble Auvergne‐Rhône‐Alpes Villeurbanne France; ^4^ Stuttgart State Museum of Natural History Stuttgart Germany; ^5^ University of Greifswald Zoological Institute and Museum Greifswald Germany; ^6^ Department of Life, Health and Environmental Sciences University of L'aquila L'aquila Italy; ^7^ Institut Universitaire de France Paris France

**Keywords:** biodiversity, energy, hyporheic zone, local species richness, New Caledonia, stream

## Abstract

At continental to global scales, energy‐related predictors relate positively to regional species richness, often explaining much of its variation in different ecosystems. However, the influence of energy on local species richness (LSR) has received much less attention. In aquatic ecosystems, LSR is predicted to increase with increasing catchment terrestrial productivity. Greater food resources resulting from increased terrestrial subsidies of nutrients and organic matter to the stream can support more individuals, thereby enabling more species to attain viable local populations, a hypothesis known as the more‐individuals hypothesis (MIH). We used generalized linear models and variance partitioning to quantify the unique and shared effects of nine local predictors and nine catchment predictors on LSR of hyporheic communities sampled at 228 sites in tropical New Caledonia streams. We found that productivity proxies, including the proportions of peridotite and bare soil in the catchment, and the normalized difference vegetation index (NDVI), correlated strongly with proxies of sediment metabolism (dissolved oxygen and pH), which in turn accounted for much of the variation in LSR. However, the positive relationship between LSR and catchment productivity, and the resulting dominance of shared effects of local and catchment predictors, were largely driven by fast‐growing taxa. Their richness and abundance increased monotonically with increasing catchment productivity. The LSR of slow‐growing taxa was largely explained by local predictors, and it showed a weak, hump‐shaped relationship to catchment productivity. The additional food resources from more productive catchments could either not be assimilated by slow‐growing taxa or were monopolized by fast‐growing taxa. Hence, other processes than those underlying the MIH, including the competitive ability of taxa to acquire available resources, likely play a role in controlling the species‐energy relationship in hyporheic invertebrate communities.

## Graphical abstract

How does the local species richness (LSR) of stream invertebrate communities relate to the terrestrial productivity of the stream catchment? Using generalized linear models and variance partitioning, we quantified the independent and joint effects of local and catchment predictors on LSR of hyporheic invertebrate communities in New Caledonia streams. We found that catchment terrestrial productivity accounted for much of the variation in LSR for fast‐growing taxa but not for slow‐growing taxa. We conclude that other factors than the availability of food resources, including the competitive ability of taxa to acquire those resources, likely play a role in controlling the productivity–energy relationship in hyporheic invertebrate communities.

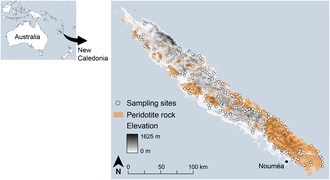

## 1 Introduction

Understanding the factors that shape local biological communities is a cornerstone of ecology (Shorrocks and Sevenster 1995; Passy et al. 2024). Since the 1990s, ecologists have recognized that the richness of local communities depends on factors that operate at scales ranging from local to regional (Ricklefs 1987). A standard approach consists in decomposing variation in local species richness (LSR) into local and landscape (region) components in order to disentangle the relative effects of factors operating at these two different spatial scales (Brown et al. 2011; Li et al. 2021). However, in many studies, the unique effects of local and landscape components are considerably smaller than their shared effects (White and Hurlbert 2010; Ricklefs and He 2016; Keil and Chase 2019). The overriding contribution of shared effects suggests that landscape factors exert a major control on local factors, which in turn influence LSR (Mouron et al. 2022). Shared effects are expected to be important in aquatic landscapes, as the terrestrial catchments provide much of the energy and matter that contribute to the heterogeneity and trophic resources of local aquatic habitats, which may ultimately determine the richness of local communities (Allan and Johnson 1997; Davies et al. 2000; Harvey and Altermatt 2019).

The idea that in‐stream habitat attributes, especially the amount of food resources available to biological communities, are strongly influenced by catchment factors was central to Hynes's eloquent plea (Hynes 1975, 4) that ‘the fertility of the valley rules that of the stream in many ways’. Inputs of nutrients from soils stimulate in‐stream primary productivity and terrestrial dissolved and particulate organic carbon sustains secondary productivity (Bartels et al. 2012; Soininen et al. 2015; Gounand et al. 2018). Greater food resources resulting from terrestrial subsidies can support higher abundances of aquatic consumers, potentially enabling more species to attain viable population sizes, a species—productive energy hypothesis known as the more‐individuals hypothesis (MIH) (Srivastava and Lawton 1998; Evans, Warren, and Gaston 2005; Storch et al. 2018). Several continental to global scale studies found that regional species richness (RSR) in diverse freshwater taxa, including fishes, amphibians, and invertebrates, increased with increasing terrestrial productivity (Oberdoff et al. 1995; Guégan et al. 1998; Tisseuil et al. 2013; Eme et al. 2015), suggesting that freshwater biodiversity may be partly controlled by terrestrial resource subsidies. In these studies, species richness and productivity were measured at extremely large grain sizes corresponding to the entire catchments of large rivers or 10,000‐km^2^ quadrats. However, comparatively fewer studies tested whether LSR of freshwater communities increased with increasing terrestrial productivity of their contributing catchment (Vinson and Hawkins 2003; Soininen and Luoto 2012; Pajunen et al. 2017), leaving unresolved the question of whether the species—productive energy relationship holds true at spatial grain size smaller than the region.

Testing the link between catchment terrestrial productivity and species richness of freshwater communities poses a number of challenges. First, terrestrial productivity often correlates positively with temperature (Brown et al. 2004; Wang et al. 2009; Šímová and Storch 2017), so that it may prove difficult to separate their effects (but see Bohdalková et al. 2021). Second, the species‐energy relationship that emerges at the level of whole communities (or clades) may result from the aggregation of taxa (or subclades) whose richness may have very different relationships with energy (Evans, Greenwood, and Gaston 2005; Buckley et al. 2010; Hurlbert and Stegen 2014). Decomposing the species‐energy relationship based on the ecological characteristics of the species that make up the communities can provide a better understanding of the mechanisms linking energy and species richness (Reed et al. 2006; Kriegel et al. 2023). Third, the majority of mechanisms proposed to explain the observed positive relationship between species richness and productivity, including the MIH, assumed that productivity influences species richness through its effect on abundance, the total number of individuals in the community (Clarke and Gaston 2006; Hurlbert and Stegen 2014; Storch et al. 2018; Furness et al. 2021). However, large‐scale studies reporting a positive correlation between freshwater RSR and terrestrial productivity could not test the above assumption because they lacked abundance data (Tisseuil et al. 2013; Eme et al. 2015).

Here, we address the aforementioned challenges by quantifying the unique and shared effects of local and catchment predictors on LSR of hyporheic invertebrate communities in tropical streams of New Caledonia. Hyporheic habitats—the water‐saturated sediments beneath and alongside the streambed through which stream water flows—display extensive geomorphic, hydrologic, and trophic linkages with their contributing catchments (Malcolm et al. 2005; Malard et al. 2017; Saup et al. 2019; Tonina and Buffington 2023). Microbial respiration and the abundance of invertebrate communities in hyporheic sediments are fueled by in‐stream production of photosynthetic organic matter and allochthonous inputs of dissolved and particulate organic matter (Strayer et al. 1997; Capderrey et al. 2013; Große et al. 2025); both sources are likely to increase with increasing catchment terrestrial productivity. Yet, hyporheic invertebrate communities are mixed assemblages of slow‐ and fast‐growing taxa that are expected to respond differently to increased organic matter supply due to their distinct requirements and competitive ability for food resources. According to the clinal model of hyporheic communities (Brunke and Gonser 1999; Capderrey et al. 2013), the species richness of fast‐growing taxa would be limited by the availability of food resources, whereas that of slow‐growing taxa would be more constrained by competition with the former.

New Caledonia shows strong spatial variation in terrestrial productivity, which is primarily determined by geology rather than temperature: nutrient‐poor and metal‐rich ultramafic soils developing on peridotite rocks have much lower productivity than non‐ultramafic soils associated with volcano‐sedimentary rocks (Isnard et al. 2016; Pillon et al. 2021). We hypothesized that catchment terrestrial productivity would exert control on LSR and made four predictions as follows. First (P1), catchment's control on in‐stream local conditions would result in significant co‐variation between local and catchment predictors (Davies et al. 2000). More specifically, dissolved oxygen and pH of hyporheic water, two local predictors, would decrease with increasing catchment productivity if terrestrial subsidies of nutrients and organic carbon control oxygen consumption and CO_2_ release linked to microbial respiration. Second (P2), if co‐variation between local and catchment predictors accounts for a major variation of LSR, then the shared effects of local and catchment predictors would be higher than their unique effects (Mouron et al. 2022). Third (P3), if increased terrestrial resource subsidies from more productive catchments influence species richness through their effect on abundance, then LSR, total abundance, and catchment productivity would correlate positively as expected under the MIH (Storch et al. 2018). Fourth (P4), the contribution of shared effects and the positive relationship of LSR to catchment productivity will be stronger for fast‐growing than slow‐growing taxa, due to their greater energy requirements to maintain viable populations and greater ability to compete for food resources (Brunke and Gonser 1999).

## 2 Materials and Methods

### 2.1 Data Collection

#### 2.1.1 Species Richness

**FIGURE 1:**
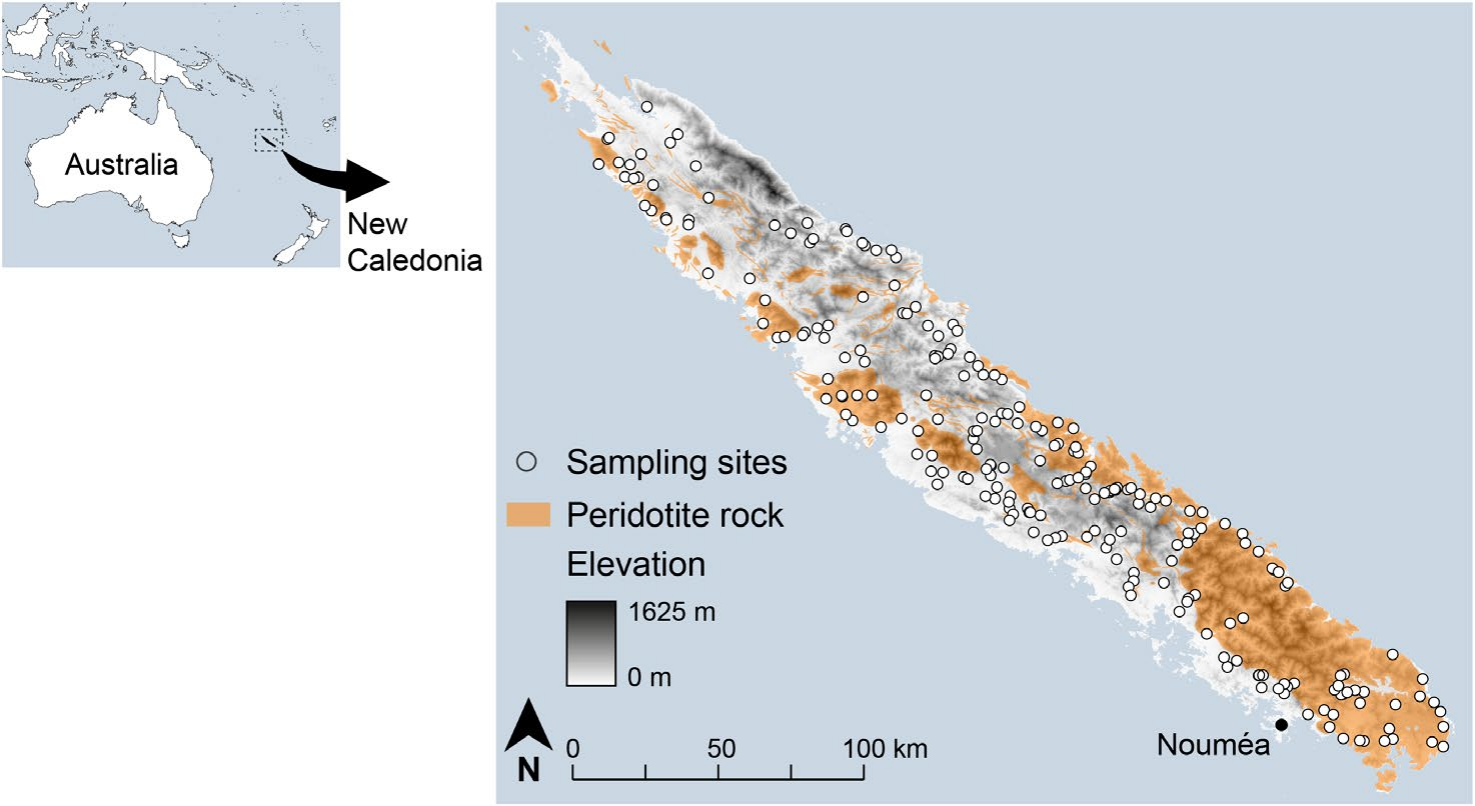
Location of hyporheic sampling sites (*n* = 228) in streams of New Caledonia.

We sampled hyporheic invertebrate communities at 228 sites on the main island of New Caledonia on a single occasion during three campaigns undertaken in November 2016, November 2017, and July 2018 (Figure 1). All samples were collected during the dry season when river flows were minimal, but over three consecutive years (61, 95, and 72 sites in 2016, 2017, and 2018, respectively, Text S1: Figure ST1.1; Table S2). Low flow stream discharge and the size of upstream catchments at sampling sites ranged from 0.1 to 3200 L.s^−1^ (median: 116 L.s^−1^) and 0.1 to 486 km^2^ (median: 30.1 km^2^), respectively (Table S2). At each site, we grouped together three replicate samples, which were collected over a distance of about 10 m. We obtained each replicate sample by pumping 10 L of water and sediment into a perforated pipe, which we hammered to a depth of 60 cm below the streambed (Boulton et al. 2003). We elutriated and filtered water and sediment through a 150‐μm mesh sieve and preserved materials remaining on the sieve in 96% ethanol. In the laboratory, we counted all obligate aquatic invertebrates in preserved material using a dissecting microscope. We defined obligate aquatic invertebrates as invertebrates that spend their entire life cycle in water (Platyhelminthes, Nematoda, Mollusca, Nemertea, Annelida, and Crustacea). We identified invertebrates to the lowest practical level, in most cases to the species level (Table S1). We distinguished between surface water species, which are found in the hyporheic zone, and obligate groundwater species, which complete their entire life cycle exclusively in groundwater habitats. We refer to the former as fast‐growing species and to the latter as slow‐growing species, since surface water species exhibit faster somatic growth rates and shorter life spans and reach reproductive maturity at younger ages than obligate groundwater species (Østbye et al. 2018; Venarsky, Niemiller, et al. 2023).

#### 2.1.2 Local and Catchment Predictors

We used methods described in detail by Mouron et al. (2022) to measure nine local predictors and nine catchment predictors (see also Text S1, Figure ST1.2, and Table S2). Local predictors were as follows: We measured dissolved oxygen, pH, and redox potential of hyporheic water as proxies of hyporheic sediment metabolism. These variables are expected to be lower in sediments receiving higher organic matter supply because of increased microbial respiration (Malard et al. 2002; Reeder et al. 2018). We measured specific conductance of hyporheic water as a proxy of water residence time in the hyporheic zone or lateral aquifers hydrologically connected to it (Cox et al. 2007). In addition to one‐time measurement of hyporheic water temperature, we used the mean annual air temperature at the sampling sites, which we extracted from the WorldClim 2 dataset (30 arc‐second resolution, Fick and Hijmans 2017), as a surrogate of mean annual hyporheic water temperature (Eme et al. 2014). Stream width was measured as a proxy for the amount of available habitat for hyporheic invertebrates. We measured stream elevation and slope at the sampling sites using a 10‐m resolution digital elevation model and a digital network of New Caledonian Streams computed from this model.

We used the above‐mentioned digital elevation model and digital stream network to delimit the upstream contributing catchment area associated with each sampling site. We computed the following catchment predictors: the areal proportion of peridotite rocks, normalized difference vegetation index (NDVI), three land cover predictors (land cover predictors 1–3, see Text S1), areal proportion of surfaces eroded by mining activities, catchment area, mean annual precipitation, and low flow specific stream discharge. Values of land cover predictor 1 increased with increasing proportion of bare soil in the catchment. We used the areal proportion of peridotite rocks, land cover predictor 1, and NDVI as proxies of catchment terrestrial productivity. Catchment productivity is expected to decrease with increasing proportions of peridotite rocks and bare soil and to increase with increasing NDVI. We retained the areal proportion of surfaces eroded by mining activities as a predictor because nickel mining, which occurs exclusively in peridotite catchments, may affect freshwater invertebrates. Opencast mining removes soil and vegetation (Bertrand and Liébault 2019), thereby reducing primary productivity of the catchment. Trace metals resulting from mining activity, more especially Ni and Cr, may have direct toxicity effects on invertebrates (Leonard and Wood 2013; Brix et al. 2017), or they can constrain food resources by constraining gross primary production in streams (Cervantes et al. 2001). We performed all geospatial analyses in *ArcGIS* 10.2.2 (Esri, Redlands, California, USA) and QGIS 3.30.3‐1 (QGIS Development Team 2023).

### 2.2 Statistical Analysis

We used Pearson's *r* correlation coefficients to test for significant co‐variation between local and catchment predictors (P1). We employed generalized linear models (GLMs) and variance partitioning to test the prediction that catchment productivity would positively affect LSR by increasing the supply of nutrients and organic carbon to the hyporheic zone (P2). For each sampling site, we calculated estimates of the total number of species as well as the number of fast‐ and slow‐growing species, at a sample coverage level of 95% using the estimateD function for abundance data of the iNEXT package (Hsieh et al. 2016). We referred to these estimates as the local species richness of the total community (LSRt), fast‐growing species (LSRf), and slow‐growing species (LSRs). We measured abundance as the number of individuals per 30 L of pumped water and sediment.

Before modeling LSRt as a function of local and catchment predictors, we calculated variance inflation factors (VIFs) to evaluate the multicollinearity within and between the two types of predictors. We normalized and included all predictors in the GLMs in their linear form and some in their quadratic form to allow for the possibility of minor nonlinearity in the relationship between LSRt and the predictors. To select the predictors to be included in their quadratic form, we modeled the relationship between LSRt and each predictor separately using generalized additive models (GAMs) with a Poisson distribution and a basis dimension (*k*) of 3. We included all predictors that showed a significant relationship with LSR (*p* < 0.05) and an effective degree of freedom (EDF) higher than 1.5 in both their linear and quadratic forms in subsequent GLMs. To account for potential overdispersion and zero‐inflation in the LSRt data, we fitted GLMs using four different distributions—Poisson, zero‐inflated Poisson, negative binomial, and zero‐inflated negative binomial—and retained the GLMs distribution with the smallest Akaike information criterion corrected for small sample size (AICc).

We used the three‐step analytical procedure described by Eme et al. (2015) and Mouron et al. (2022) to quantify the variation in LSRt explained by each predictor as well as the unique and shared effects of catchment and local predictors. In step 1, we assessed the variation explained by each of the two predictor types separately and quantified the importance of each predictor within its type using multi‐model inferences (Burnham and Anderson 2002). For each predictor type, we ran all possible GLMs and retained only those models whose difference in AICc with the model showing the lowest AICc was ≤ 5. We measured the relative importance of each predictor within its type using two indicators: the sum of AICc weights of models in which the predictor occurred and the proportion of deviance reduction due to a predictor. We retained those predictors with an AICc weight ≥ 0.7 to build the local and catchment models.

In step 2, we assessed the variation in LSRt explained by the two types of predictors in a joint model containing the predictors retained at step 1 in the local and catchment models. As in step 1, we used multi‐model inference and the same two indicators to quantify the importance of each predictor in the joint model. We performed the analyses in steps 1 and 2 on a total dataset comprising 228 samples collected in 2016, 2017, and 2018. However, we repeated these analyses separately for each year to test whether the predictors controlling LSR differed when analyzing the total dataset compared to analyzing each year separately.

In step 3, we performed variance partitioning (Chevan and Sutherland 1991; Legendre and Legendre 2012) using Nagelkerke's pseudo *R* ^2^ (Nagelkerke 1991) to quantify the unique and shared effects of the two predictor types.

To test the prediction that shared effects would be stronger for fast‐growing taxa than slow‐growing taxa (P4), we reiterated the full modeling procedure described above for LSRt using LSRf and LSRs as response variables. Then, to test for positive relationships among LSRt, total abundance, and catchment productivity (P3), we followed the framework proposed by (Storch et al. 2018) and used linear models (LMs) on ln (*x* + 1) transformed species richness and abundance data for the total community. We used the areal proportion of peridotite rocks and NDVI as proxies of catchment productivity. We also employed LMs using LSR and abundance of slow‐ and fast‐growing taxa to test the prediction that positive LSR‐catchment productivity relationships would be stronger for fast‐growing than slow‐growing taxa (P4).

To tease apart the potential effect of the areal proportion of surfaces eroded by mining activities on LSR from that of the areal proportion of peridotite rocks, we performed GLMs on a subset of sites whose catchments had an areal proportion of peridotite rocks ≥ 85% (*n* = 63 sites). We modeled the relationship between LSRt, LSRf, and LSRs and each predictor using GAMs. We included all significant predictors as well as the areal proportion of surfaces eroded by mining activities in a joint model and quantified the importance of each predictor in the joint model.

All statistical analyses were conducted in R 4.4.1 (R Core Team 2024). GLMs were performed using the glmmTMB package (Brooks et al. 2017), GAMs with the mgcv package (Wood 2017), and multi‐model inference with the MuMIn package (Bartoń 2024). We computed VIFs using the AED script from (Zuur et al. 2009), and variance partitioning for GLMs with a simple set of equations following Legendre and Legendre (2012).

## 3 Results

### 3.1 Co‐Variation Between Local and Catchment Predictors (P1)

**FIGURE 2:**
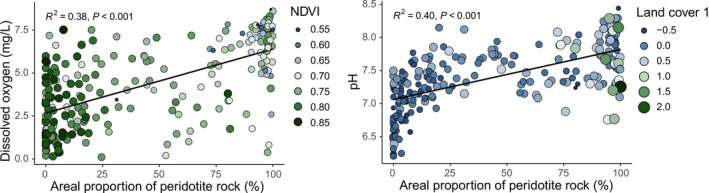
Left panel: The relationships between dissolved oxygen concentrations in hyporheic water at 228 stream sites, the areal proportion of peridotite rock in the contributing catchments, and the normalized difference in the vegetation index (NDVI) in the catchments. Right panel: The relationship between pH of hyporheic water, the areal proportion of peridotite rock, and landcover predictor 1, a proxy for the proportion of bare soil in the catchments.

Dissolved oxygen and pH of hyporheic water were positively correlated (*r* = 0.63), and both increased significantly with the areal proportion of peridotite rocks in the catchment (*r* = 0.63 and *r* = 0.62, respectively) (Figure 2, Table S3). The latter was negatively correlated to NDVI (*r* = −0.60) and positively correlated (*r* = 0.48) to the areal proportion of bare soils in the catchment (land cover predictor 1). The VIFs of most predictors were < 4, except for dissolved oxygen, the proportion of peridotite, NDVI, and land cover predictor 1, due to multicollinearity among these predictors (Table S4).

### 3.2 Influence of Local and Catchment Predictors on LSR (P2)

We identified 17,650 specimens belonging to 118 fast‐growing taxa and 65 slow‐growing taxa, of which 86 were potentially new to science (Table S1). The average local species richness (±standard deviation) was 9.3 ± 5.5, 7.5 ± 5.8, and 2.3 ± 1.6 taxa for the total community (LSRt), fast‐growing taxa (LSRf), and slow‐growing taxa (LSRs), respectively.

**FIGURE 3:**
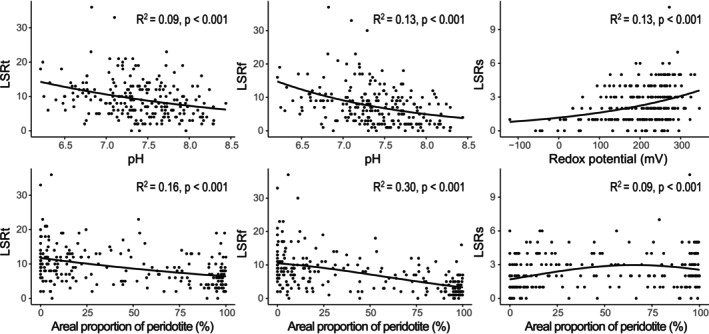
Relationships between the local species richness (LSR) of permanent aquatic invertebrates in the hyporheic zone of New‐Caledonia streams (*n* = 228 sites) and the pH, redox potential of hyporheic water, and the areal proportion of peridotite in the contributing catchments. LSRt, LSRf, and LSRs correspond to the local species richness of the total community, fast‐growing species, and slow‐growing species, respectively. Relationships are fitted using the generalized linear model with the smallest AICc.

We retained the negative binomial distribution (i.e., the one with the smallest AICc) in GLMs for LSRt and LSRf and the Poisson distribution for LSRs (Table S5). Of nine local predictors, three, four, and five were retained (AICc weight ≥ 0.7) in local models for LSRt, LSRf, and LSRs, respectively (Table 1, Table S6, Figure S1). pH explained the highest proportion of model variation for LSRt and LSRf, whereas redox potential was the most explanatory predictor for LSRs. LSRt and LSRf decreased with increasing pH, and LSRs increased with increasing redox potential (Figure 3).

**TABLE 1:** Summary results of generalized linear models for testing the effects of local and catchment predictors on local species richness of permanent aquatic invertebrates in the hyporheic zone of New‐Caledonia streams. For local and catchment models, we retained only those predictors with an Akaike information criterion corrected for small sample size (AICc) weight > 0.7 (See Figure S1).

Local species richness (LSR)	Model	Predictors	AICc weight	Explained variation (%)	Coefficient	*p*
Community (LSRt)	Local	Intercept			2.182	< 0.001
		pH	1	1.57	−0.186	**< 0.001**
		Elevation^2^	0.97	0.56	−0.041	**0.0102**
		Water temperature^2^	0.86	0.3	0.065	**0.0441**
	Catchment	Intercept			2.156	**< 0.001**
		Peridotite	1	2.88	−0.194	**< 0.001**
		Land cover 1	0.98	0.26	−0.145	**0.0034**
		Land cover 1^2^	0.94	0.44	0.040	**0.0163**
	Joint	Intercept			2.139	**< 0.001**
		Peridotite	1	2.88	−0.140	**0.0058**
		Land cover 1	1	0.63	−0.151	**0.0022**
		Land cover 1^2^	1	0.06	0.043	**0.0113**
		Elevation^2^	0.79	0.36	−0.035	**0.0252**
		Water temperature^2^	0.48	0.13	0.045	0.1485
		pH	0.39	0.12	−0.058	0.2024
Fast‐growing species (LSRf)	Local	Intercept			2.174	**< 0.001**
		pH	1	2.36	−0.248	**< 0.001**
		Dissolved oxygen^2^	0.97	0.4	−0.173	**0.0033**
		Elevation^2^	0.92	0.61	−0.049	**0.0141**
		Redox potential	0.78	0.39	−0.117	**0.0226**
	Catchment	Intercept			2.133	**< 0.001**
		Peridotite	1	5.88	−0.470	**< 0.001**
		Peridotite^2^	1	0.18	−0.237	**0.0053**
		Land cover 1	1	0.12	−0.356	**< 0.001**
		Land cover 1^2^	0.98	0.6	0.072	**< 0.001**
		NDVI	0.98	0.24	−0.285	**0.0044**
		Catchment area^2^	0.95	0.21	−0.072	**0.0044**
		Catchment area	0.88	0.3	0.166	**0.0179**
		Discharge	0.8	0.39	0.136	**0.0249**
	Joint	Intercept			2.229	**< 0.001**
		Peridotite	1	5.88	−0.415	**< 0.001**
		Land cover 1	1	0.59	−0.405	**< 0.001**
		Land cover 1^2^	1	0.1	0.079	**< 0.001**
		NDVI	1	0.02	−0.322	**0.0012**
		Peridotite^2^	0.94	0.42	−0.213	**0.0151**
		Catchment area^2^	0.94	0.21	−0.071	**0.0041**


		Discharge	0.91	0.26	0.143	**0.0226**
		Elevation^2^	0.89	0.47	−0.042	**0.0239**
		Catchment area	0.81	0.37	0.160	**0.0211**
		Dissolved oxygen^2^	0.6	0.23	−0.093	0.0972
pH		0.3	0.06	−0.047	0.4375
Redox potential	0.2	0.02	−0.023	0.6264
Slow‐growing species (LSRs)	Local	Intercept			0.744	**< 0.001**
		Redox potential	1	3.56	0.269	**< 0.001**
		Stream width	0.99	0.59	0.104	**0.0101**
		Mean annual air temperature^2^	0.99	0.23	0.099	**0.0062**
		Elevation^2^	0.96	1.33	−0.059	**0.0373**
		Specific conductance	0.92	0.67	0.103	**0.0184**
	Catchment	Intercept			1.064	**< 0.001**
		Peridotite	1	1.68	0.231	**< 0.001**
		Peridotite^2^	1	0.78	−0.255	**0.0020**
		Precipitation	0.99	0.77	0.111	**0.0122**
	Joint	Intercept			0.977	**< 0.001**
		Redox potential	1	3.56	0.235	**< 0.001**
		Elevation^2^	0.97	0.31	−0.068	**0.0235**
		Peridotite^2^	0.96	0.2	−0.236	**0.0084**
		Mean annual air temperature^2^	0.95	1.54	0.099	**0.0169**
		Peridotite	0.87	1.05	0.130	**0.0225**
		Stream width	0.78	0.53	0.087	**0.0383**
		Specific conductance	0.5	0.17	0.076	0.1308
		Precipitation	0.45	0.25	0.072	0.1626

*Note:* The joint models include all predictors retained in the local and catchment models. Proportions of explained variation (i.e., proportion in reduction of deviance), coefficient parameters and *p* values are from the local and catchment models with the lowest AICc and from the joint model including all the predictors retained in the best local and catchment models. In bold, significant *p* values for the predictors (*p* < 0.05). The superscript next to the predictor's names indicate the quadratic form.

Of nine catchment predictors, two, five, and two were retained in catchment models for LSRt, LSRf, and LSRs, respectively (Table 1, Table S6, Figure S1). The areal proportion of peridotite explained the highest proportion in LSR variation in all three catchment models. The proportion of bare soil (i.e., land cover 1) was the second most explanatory predictor of LSRt and LSRf, followed by NDVI for LSRf (Table 1, Figure S2). LSRt and LSRf decreased with increasing proportion of peridotite, whereas LSRs showed a hump‐shaped relationship to that predictor (Figure 3).

The areal proportions of peridotite and bare soils remained the first and second most explanatory predictors in joint models, respectively, for LSRt and LSRf, whereas pH and DO were no longer significant (Table 1). Redox potential explained by far the highest proportion of joint model variation for LSRs. We obtained similar results when analyzing each year separately: peridotite in 2016 and 2018, and bare soils in 2018 were the first explanatory predictors in joint models for LSRt (see Text S2: Table ST2.1).

The areal proportion of surfaces eroded by mining activities was not retained in any of the catchment models performed on the full data set (*n* = 228 sites) to explain LSRt, LSRf, and LSRs. That predictor had one of the lowest AICc weights (< 0.18) among all the predictors (Figure S1). It was also not significant in joint models performed on the subset of sites with an areal proportion of peridotite rocks ≥ 85% (*n* = 63 sites, see Text S3: Tables ST3.1, ST3.2, ST3.3, and Figure ST3.4).

### 3.3 Unique and Shared Effects of Local and Catchments Predictors on LSR (P2 & P4)

**FIGURE 4:**
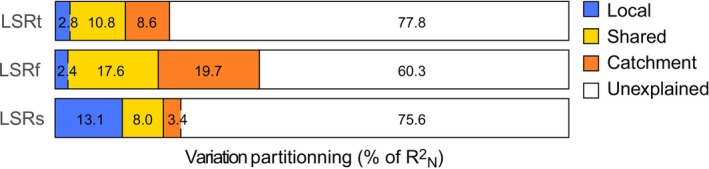
Proportions of variation in the local species richness of permanent aquatic invertebrates in the hyporheic zone of New‐Caledonia streams explained by the unique and shared effects of local and catchment predictors. Variation partitioning was performed using Nagelkerke's pseudo R^2^_N_. LSRt, LSRf, and LSRs correspond to the local species richnesses of the total community, fast‐growing species, and slow‐growing species, respectively.

Local and catchment predictors accounted for 22.2%, 39.7% and 24.5% of the variation in LSR in the total community, fast‐growing taxa, and slow‐growing taxa, respectively (Figure 4). Variance partitioning revealed substantial differences in the proportion of variation in LSR explained by the shared and unique effects of local and catchment predictors between fast‐ and slow‐growing taxa. In fast‐growing taxa and the total community, shared effects explained 7.3 and 3.9 times more variation than the unique effects of local predictors, respectively (Figure 4). In contrast, in slow‐growing taxa, the unique effects of local predictors largely exceeded the shared effects of predictors as well as the unique effects of catchment predictors.

### 3.4 Relationships Among LSR, Abundance and Catchment Productivity (P3 & P4)

**FIGURE 5:**
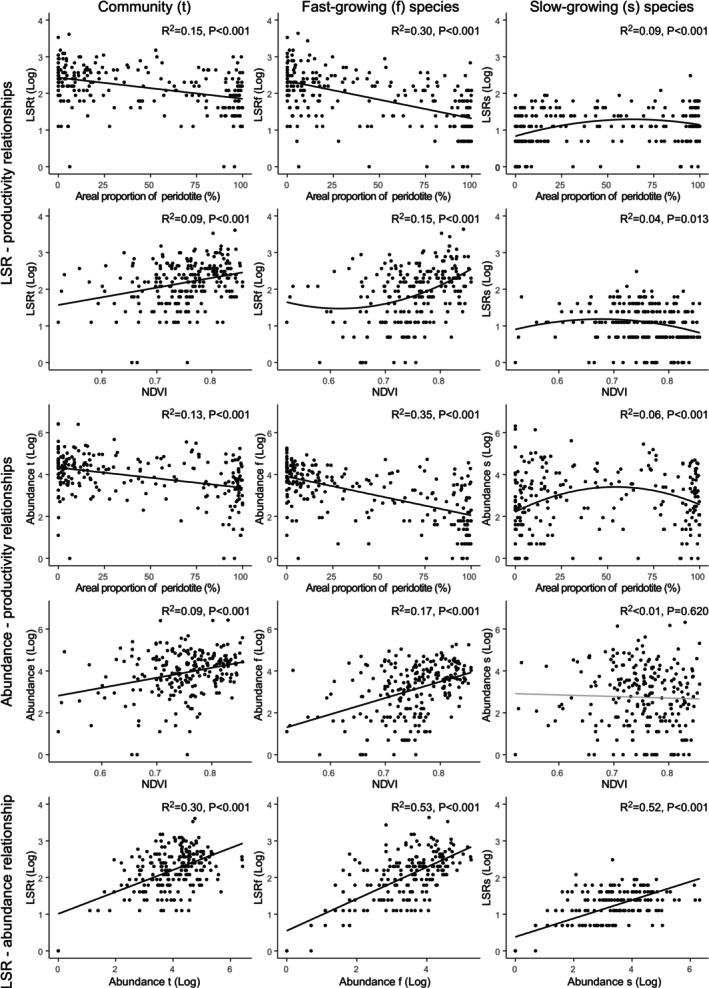
Relationships among local species richness (LSR), catchment productivity, and abundance. The areal proportion of peridotite rock and the normalized difference in the vegetation index (NDVI) are surrogates for terrestrial productivity of contributing catchments. LSRt, LSRf, and LSRs correspond to the local species richness of the total community, fast‐growing species, and slow‐growing species, respectively.

We found significant, increasing relationships between LSR and catchment productivity (when using either the proportion of peridotite or NDVI), between abundance and catchment productivity, as well as between LSR and abundance for the total community and fast‐growing taxa (Table 2, Figure 5, Table S7). Relationships explained a higher proportion of variance (higher *R* ^2^) for fast‐growing taxa than for the total community, and when peridotite was used as a proxy for productivity rather than NDVI. Productivity explained abundance better than LSR for fast‐growing taxa.

**TABLE 2:** Summary of the relationships between local species richness (LSR), abundance and catchment productivity.

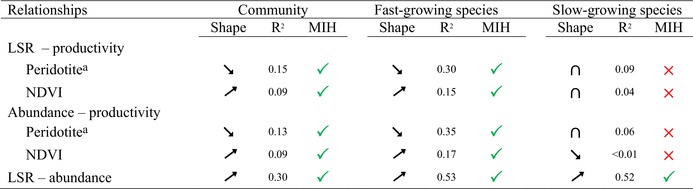

*Note:* All relationships are statistically significant (*p* < 0.05) except for the relationship between the abundance of slow‐growing species and NDVI (see Figure 5). Up arrows, down arrows and intersection symbols represent positive, negative and hump‐shaped relationships, respectively. Green tick marks and red crosses indicate support for, and rejection of, the more‐individuals hypothesis (MIH), respectively.

^a^ Catchment productivity varies inversely with the areal proportion of peridotite rocks in the catchment.

We found weak (*R* ^2^ ≤ 0.09), hump‐shaped relationships between LSR and catchment productivity, and between abundance and the proportion of peridotite for slow‐growing taxa (Table 2, Figure 5, Table S7). The abundance–NDVI relationship was not significant for slow‐growing taxa.

## 4 Discussion

The influence of energy‐related factors on species richness has long been recognized (Wright 1983). However, there is still ongoing discussion as to whether productive energy (e.g., net primary productivity), solar energy (e.g., temperature, potential evapotranspiration), or a combination of solar energy and water availability (e.g., precipitation) is the most important factor (Brown et al. 2004; Evans, Greenwood, and Gaston 2005; E. O'Brien 1998; Vetaas et al. 2019). Although the species‐energy relationship depends on spatial scale, it has essentially been studied in the context of continental‐to‐global patterns of species richness, using RSR as a dependent variable (Hawkins et al. 2003; Tisseuil et al. 2013; Coelho et al. 2025). The dependence of LSR on energy‐related factors has received much less attention, particularly in aquatic ecosystems where quantifying the productive energy available to the biota requires integrating the flow of matter and energy from adjacent terrestrial ecosystems (Soininen and Luoto 2012).

This study is the first to model variation in LSR of hyporheic invertebrate communities across an entire region using a combination of catchment and local predictors. The results support the hypothesis that LSR is indirectly controlled by catchment terrestrial productivity, which determines the productive energy available for local communities to convert into biomass. Compared to catchment productivity, neither temperature nor water availability appeared to be paramount. The gradient in catchment productivity was driven by geology and therefore decoupled from temperature. The role of water (e.g., precipitation) as a regulator of energy is probably more applicable to plants than to animals, especially aquatic animals (Vetaas et al. 2019). We found support for the predictions of the more‐individuals hypothesis (MIH, Srivastava and Lawton 1998, Storch et al. 2018), suggesting that variation in LSR is driven by variation in terrestrial resource subsidies through its effect on the total number of individuals in the community. Increased abundance resulting from higher resource subsidies allows a greater number of species to maintain locally viable populations. The indirect positive relationship between catchment productivity and LSR, and resulting dominance of shared effects of local and catchment predictors, was largely driven by fast‐growing taxa. The richness and abundance of slow‐growing taxa decreased at the upper end of the productivity gradient, potentially because they were outcompeted by fast‐growing taxa. Below, we examine each of the four predictions made under the hypothesis that catchment productivity controls LSR by regulating the number of trophic resources available to hyporheic invertebrate communities.

### 4.1 Co‐Variation Between Local and Catchment Predictors (P1)

The idea that catchment characteristics, especially geology and climate, exert a major control on in‐stream local conditions is a central tenet of freshwater ecology (Hynes 1975; Frissell et al. 1986; Fausch et al. 2002). Many studies showed that stream sites retained the biophysical imprints of the catchment from which they received sediment, water, organic matter, and nutrients (Richards et al. 1996; Davies et al. 2000; Mouron et al. 2022; Zhu et al. 2022). According to our first prediction (P1), we found that hyporheic sediment metabolism decreased significantly as catchment productivity decreased. DO, pH, and redox potential of hyporheic water increased as the proportion of peridotite rocks in the catchment area increased. The latter correlated negatively with NDVI and positively with the proportion of bare soil in the catchment. Our finding of a negative relationship between hyporheic sediment metabolism and the proportion of peridotite rocks is consistent with earlier studies showing that the biochemical oxygen demand of surface water in New Caledonian streams was significantly lower in peridotite catchments than in non‐peridotite catchments (Mary 1999). Also, we found that the content of particulate organic carbon in stream hyporheic sediment of New Caledonia was, on average, 3.2 times lower in peridotite catchments (4.1 ± 4.1 mg C per L of pumped water, *n* = 15) than in non‐peridotite catchments (13.1 ± 10.6, *n* = 15) (Mouron S. et al. unpublished data). We acknowledge that measuring hyporheic sediment respiration would have provided a better predictor of the flux of organic carbon available to invertebrates than DO because the latter is also influenced by the transit time of water within the sediment (Malard and Hervant 1999).

The higher correlations between proxies of sediment metabolism (e.g., DO and pH) and the proportion of peridotite rocks suggest that the latter is a better indicator of the number of trophic resources supplied to the stream by the watershed than the sole estimate of the amount of above‐ground biomass provided by NDVI. First, nutrient‐poor soils that develop on peridotite rocks may not only deliver fewer particulate and dissolved organic carbon to the stream, but also fewer nutrients to sustain algal primary production. Yet, allochthonous organic carbon and the algal biomass that is incorporated into the streambed sediment fuel microbial and invertebrate communities in the hyporheic zone (Kaplan and Newbold 2000; Serchan et al. 2024). Organic matter is a key regulator of hyporheic invertebrate communities: their abundance and richness are expected to increase with an increased supply of organic matter until dissolved oxygen (DO) eventually becomes limiting due to microbial respiration (Strayer et al. 1997; Marmonier et al. 2023). Second, gross primary production in streams of peridotite catchments may be constrained by the presence of easily leachable toxic metals, such as Cr (VI), in suspended and streambed sediments (Cervantes et al. 2001; Gunkel‐Grillon et al. 2014). The high trace metal content of stream sediment in peridotite catchments, more especially Ni and Cr (Gunkel‐Grillon et al. 2014; Boula et al. 2022), may also have direct toxicity effects on stream invertebrates (Leonard and Wood 2013). Hence, metal toxicity might covary positively with catchment productivity to constrain local species richness, but that covariation was not taken into consideration in the present study.

In addition to the relationship between catchment productivity and hyporheic sediment metabolism, we identified two other significant correlations between catchment and local predictors that could potentially influence LSR. First, the amount of alluvial habitat available for hyporheic invertebrates, estimated by measuring the active channel width of alluvial streams, was positively related to catchment area (*r* = 0.56, see Bertrand and Liébault (2019) for a detailed analysis of that relationship for New Caledonian streams). Second, low flow stream discharge relative to catchment area—a proxy of the permanence of hyporheic habitat during the dry season—increased with increasing areal proportion of peridotite (*r* = 0.56) because groundwater inputs from multilayer peridotite aquifers sustained stream flow during prolonged periods of no rainfall (Romieux and Wotling 2016; Jeanpert 2017).

### 4.2 Unique and Shared Effects of Local and Catchments Predictors on LSR (P2)

Our second prediction (P2) that the shared effects of local and catchment predictors on LSR would surpass their unique effects received mixed support. Shared effects for the total community (LSRt) and fast‐growing species (LSRf) were substantially higher than the unique effect of local predictors but only slightly higher (LSRt) or lower (LSRf) than the unique effect of catchment predictor. The substantial contribution of shared effects was due to the covariation between proxies of catchment productivity (i.e., the proportions of peridotite and bare soil, and NDVI) and proxies of sediment metabolism (i.e., pH and DO). The latter explained most of the variation in LSRt and LSRf in local models but they were no longer significant in joint models because of their correlation with proxies of catchment productivity. Shared effects accounted for an overwhelming proportion of variation in aquatic species diversity in most studies that used variance partitioning to quantify the importance of local and catchment/landscape factors (Sandin and Johnson 2004; Kuglerová et al. 2015; Cai et al. 2017; Li et al. 2021). We argue that shared effects would necessarily confound attempts at trying to partition the relative importance of unique effects of local and catchment/landscape predictors on local diversity of aquatic communities because the latter is driven by local factors occurring under the environmental conditions’ characteristic of the surrounding landscape.

Following Soininen et al. (2015), we propose three explanations for the important unique contribution of catchment predictors, especially catchment productivity, on variation in LSRt and LSRf. First, they may reflect the effects of some local predictors that were not measured, especially the standing crop of organic matter in the hyporheic zone. Second, they may provide more robust time‐averaged proxies of in‐stream productivity than one‐time measurements of sediment metabolism. Third, catchment productivity may be a better explanatory predictor of LSR than the amount of food resources available locally to communities if dispersal among local communities within a catchment tends to reduce differences in LSR between productive and unproductive sites (Chase and Ryberg 2004; Soininen et al. 2015). A direct outcome of the importance of unique effects of catchment and its shared effects with local predictors is that local species richness can potentially be predicted reliably from catchment predictors alone.

### 4.3 Relationships Among LSR, Abundance and Catchment Productivity (P3)

Our results for LSRt and LSRf followed the predictions of MIH as formalized by Storch et al. (2018), namely a positive relationship between productive energy and total abundance, a tight positive relationship between abundance and species richness, and for fast‐growing taxa, a species richness‐productivity relationship weaker than the abundance‐productivity relationship. The MIH is more relevant, and therefore more likely to be validated at large spatial scales, which encompass the entirety of species populations, than at small spatial scales (Storch et al. 2018). At the latter, population viability should be less energy‐dependent, as it is also influenced by immigration from neighboring communities. However, immigration might have played a lesser role in the present study, given that many sites belonged to independent watersheds draining into the ocean. Dispersal between watersheds is expectedly low for permanent aquatic taxa, at least in comparison with aquatic insects that have an aerial dispersal stage (Bohonak and Jenkins 2003).

More generally, the ability of environmental predictors to explain species richness, including energy‐related ones, decreases with decreasing spatial grain size of analysis (O'Brien 2006; Field et al. 2009; Belmaker and Jetz 2011). Indeed, we found that proxies of catchment productivity explained at best 16%–30% of the variation in LSRf, whereas energy‐related factors typically explained > 50% of the variation in RSR in studies of broad‐scale richness patterns (Currie 1991; Wright et al. 1993; Hawkins et al. 2003; Field et al. 2009; Tisseuil et al. 2013). Our results are consistent with those of Hawkins et al. (2003) and Soininen and Luoto (2012), who showed that the richness of local stream insect genera worldwide and plankton in Finnish lakes increased with increasing NDVI values of surrounding landscapes, respectively. They also corroborate the findings of earlier studies conducted in New Caledonia, that peridotite catchments exhibited the lowest local species richness in benthic and hyporheic communities (Mary and Marmonier 2000; Mary 2002). Although LSR is low in peridotite catchments, several aquatic taxa have successfully diversified and exhibit high levels of micro‐endemism (Espeland and Johanson 2010; Zielske and Haase 2015; Nattier et al. 2017).

Using a subset of sites, we explored the possibility that mining activity may have exacerbated the geologically determined richness gradient. Mining in peridotite catchments could further reduce the supply of terrestrial organic carbon per unit of stream habitat through the combined effect of deforestation and widening of alluvial stream corridors (Bertrand and Liébault 2019). However, we found no evidence that mining activity reduced local species richness in these catchments. Even if mining activity were to make peridotite catchment rivers more oligotrophic, it would prove difficult to detect the effect of mining‐induced oligotrophy on local species richness since local communities in peridotite catchment rivers are species‐poor and dominated by slow‐growing species with low energy requirements.

### 4.4 Differential Responses of Fast‐ and Slow‐Growing Taxa to Catchment Productivity (P4)

We found strong empirical support for our fourth prediction (P4) that the positive influence of catchment productivity would be stronger for fast‐growing taxa than for slow‐growing taxa. Our results are consistent with a number of studies showing that productivity‐richness relationships varied among taxa as a function of their functional traits (Evans, Warren, and Gaston 2005; Reed et al. 2006; McClain et al. 2018, 2023; Kriegel et al. 2023). Increase in catchment productivity resulted in an increase in individuals and species only in fast‐growing taxa. This increase would have probably been even more pronounced if we had considered insect larvae, which represent an important component of invertebrate hyporheic communities. However, it is nearly impossible to identify insect larvae to species level because most of them are early instar larvae. On the contrary, the variation in LSRs was largely explained by local predictors indicating that catchment characteristics, including productivity, had little direct and indirect influence on LSR of slow‐growing taxa compared to fast‐growing taxa. Indeed, we found a weak, hump‐shaped relationship between LSRs and catchment productivity. Our results suggest that the additional food resources from more productive catchments could either not be assimilated by slow‐growing taxa or they were monopolized by fast‐growing taxa. The latter seems more likely for two reasons. First, the richness of slow‐growing taxa is known to increase monotonically with increasing terrestrial productivity in subterranean ecosystems where they are numerically dominant (Culver et al. 2006; Eme et al. 2015). Second, several studies that investigated the vertical distribution of hyporheic invertebrate communities suggested that the occurrence of slow‐growing taxa in the upper resource‐rich layers of sediment was restricted by interference competition with fast‐growing taxa, whereas food limitation restricted the colonization of deeper sediment layers by the latter (Brunke and Gonser 1999; Datry et al. 2005; Capderrey et al. 2013). In cave streams, surface invertebrates are more capable of monopolizing and assimilating surpluses of food resources than groundwater invertebrates, probably because they have higher metabolic activity and growth rate (Venarsky et al. 2018; Venarsky, Simon, et al. 2023). Indeed, Mermillod‐Blondin et al. (2025) showed that surface water isopods had higher metabolism and processed sources of organic carbon at higher rates than groundwater isopods. Our results also suggest evolutionary differences in the ability of taxa to process organic matter may explain the shift in the proportion of slow‐fast trait taxa and individuals in local hyporheic invertebrate communities along a gradient of catchment productivity. Fast‐ and slow‐growing taxa dominate the communities in high‐ and low‐productivity catchments, respectively. As observed in other studies (McClain et al. 2016, 2023; Furness et al. 2021), processes other than those underlying the MIH, including the competitive ability of taxa to acquire available resources, are likely important in controlling the species‐energy relationship in hyporheic invertebrate communities. A better understanding of these processes is crucial if we are to predict changes in the richness and composition of communities in response to predicted climate change. Climate change predictions for 2070 under three different climate models and emission scenarios anticipate an increase in mean annual air temperature of 2°C (ranges across models and scenarios: 1.3°C–2.5°C) and a decrease in annual precipitation of 50 mm (−172 to +77 mm) in New Caledonia (Pouteau and Birnbaum 2016). From our current understanding of the species‐productivity relationship, we suggest that the decrease in catchment productivity that may result from increased thermal stress and/or altered water availability in terrestrial plants would decrease LSR and increase the proportion of slow‐growing taxa in hyporheic communities.

## Author Contributions

**Samuel Mouron:** conceptualization (equal), data curation (equal), formal analysis (lead), writing – original draft (equal), writing – review and editing (equal). **Yannick Dominique:** data curation (equal), project administration (equal), supervision (equal), writing – original draft (equal), writing – review and editing (equal). **David Eme:** formal analysis (equal), writing – original draft (equal), writing – review and editing (equal). **Nina Tombers:** data curation (equal), writing – original draft (equal), writing – review and editing (equal). **Diana M. P. Galassi:** data curation (equal), writing – original draft (equal), writing – review and editing (equal). **Pierre Marmonier:** data curation (equal), writing – original draft (equal), writing – review and editing (equal). **Michel Lafont:** data curation (equal), writing – original draft (equal), writing – review and editing (equal). **Colin Issartel:** data curation (equal), writing – original draft (equal), writing – review and editing (equal). **Christophe J. Douady:** conceptualization (equal), data curation (equal), project administration (equal), supervision (equal), writing – original draft (equal), writing – review and editing (equal). **Florian Malard:** conceptualization (equal), data curation (equal), formal analysis (equal), project administration (equal), supervision (equal), writing – original draft (equal), writing – review and editing (equal).

## Funding

This work was supported by Provinces Sud et Nord Gouvernement de la Nouvelle Calédonie. Association Nationale de la Recherche et de la Technologie, Cifre ANRT n° 2022/0477. Biodiversa+, Project DarCo, GA no. 101052342. Agence Nationale de la Recherche, ANR‐17‐EURE‐0018. Ministère des Outre‐Mers.

## Conflicts of Interest

The authors declare no conflicts of interest.

## Supporting information


**Text S1:** Predictor measurement methods.
**Text S2:** Model results when analyzing each year separately.
**Text S3:** Model results for the subset of sites with areal proportion of peridotite rocks ≥ 85%.
**Table S1:** Sample—species data.
**Table S2:** Environmental predictors and response variables.
**Table S3:** Pearson's correlation coefficients among predictors.
**Table S4:** Variance inflation factors.
**Table S5:** Selection of model distributions.
**Table S6:** Results of generalized additive models (GAMs) to assess nonlinearity.
**Table S7:** Model results for the relationships among LSR, abundance and catchment productivity.
**Figure S1:** AICc weights of predictors.
**Figure S2:** Relationships between LSR and local and catchment predictors.

## Data Availability

All data and scripts for data analysis are available at https://zenodo.org/records/17083758.
