# Supplementary material for: Catchment Productivity Controls Local Species Richness of Hyporheic Invertebrate Communities in Tropical New Caledonia Streams
Source: Ecol Evol. 2025 Dec 23;15(12):e72798. doi: 10.1002/ece3.72798 (PMC12723724; doi:10.1002/ece3.72798)
Supplement: Supplementary file 1 — Text S1: Predictor measurement methods. Text S2: Model results when analyzing each year separately. Text S3: Model results for the subset of sites with areal proportion of peridotite rocks ≥ 85%. Table S1: Sample—species data. Table S2: Environmental predictors and response variables. Table S3: Pearson's correlation coefficients among predictors. Table S4: Variance inflation factors. Table S5: Selection of model distributions. Table S6: Results of generalized additive models (GAMs) to assess nonlinearity. Table S7: Model results for the relationships among LSR, abundance and catchment productivity. Figure S1: AICc weights of predictors. Figure S2: Relationships between LSR and local and catchment predictors. [file ECE3-15-e72798-s001.docx]

Ecology and Evolution

Supporting Information for:

Catchment productivity controls local species richness of hyporheic invertebrates in tropical New Caledonia streams

Samuel Mouron | Yannick Dominique | David Eme | Nina Tombers | Diana M. P. Galassi | Pierre Marmonier | Michel Lafont | Colin Issartel | Christophe J. Douady | Florian Malard

Table of contents:

| **Supplementary text 1**: Predictor measurement methods | Page 02 |
| --- | --- |
| Supplementary text 2: Model results when analyzing each year separately | Page 06 |
| **Supplementary text 3**: Model results for the subset of sites with areal proportion of peridotite rocks ≥ 85 % | Page 07 |
| **Table S1**: Sample – species data | Page 12 |
| **Table S2**: Environmental predictors and response variables | Page 13 |
| **Table S3**: Pearson's correlation coefficients among predictors | Page 14 |
| **Table S4**: Variance inflation factors | Page 15 |
| **Table S5**: Selection of model distributions | Page 16 |
| **Table S6**: Results of generalized additive models (GAMs) to assess nonlinearity | Page 17 |
| **Table S7**: Model results for the relationships among LSR, abundance and catchment productivity | Page 19 |
| **Figure S1**: AICc weights of predictors | Page 20 |
| **Figure S2**: Relationships between LSR and local and catchment predictors | Page 21 |
| **Codes for analyses** | Page 22 |

# Supplementary text 1: Predictor measurement methods

Sampling sites

We sampled hyporheic invertebrate communities at 228 sites on the main island of New Caledonia on a single occasion during three campaigns undertaken in November 2016, November 2017 and July 2018 (Figure ST1.1). All samples were collected during the dry season when river flows were minimal, but over three consecutive years (61, 95, and 72 sites in 2016, 2017 and 2018, respectively). Each year, we sampled peridotite and non-peridotite catchments in order to avoid confounding inter-annual variability in local species richness (if any) with productivity predictors (peridotite, bare soils and NDVI).

Figure ST1.1: Location and distribution across year (2016, 2017, and 2018) of hyporheic sampling sites (n = 228) in streams of New Caledonia


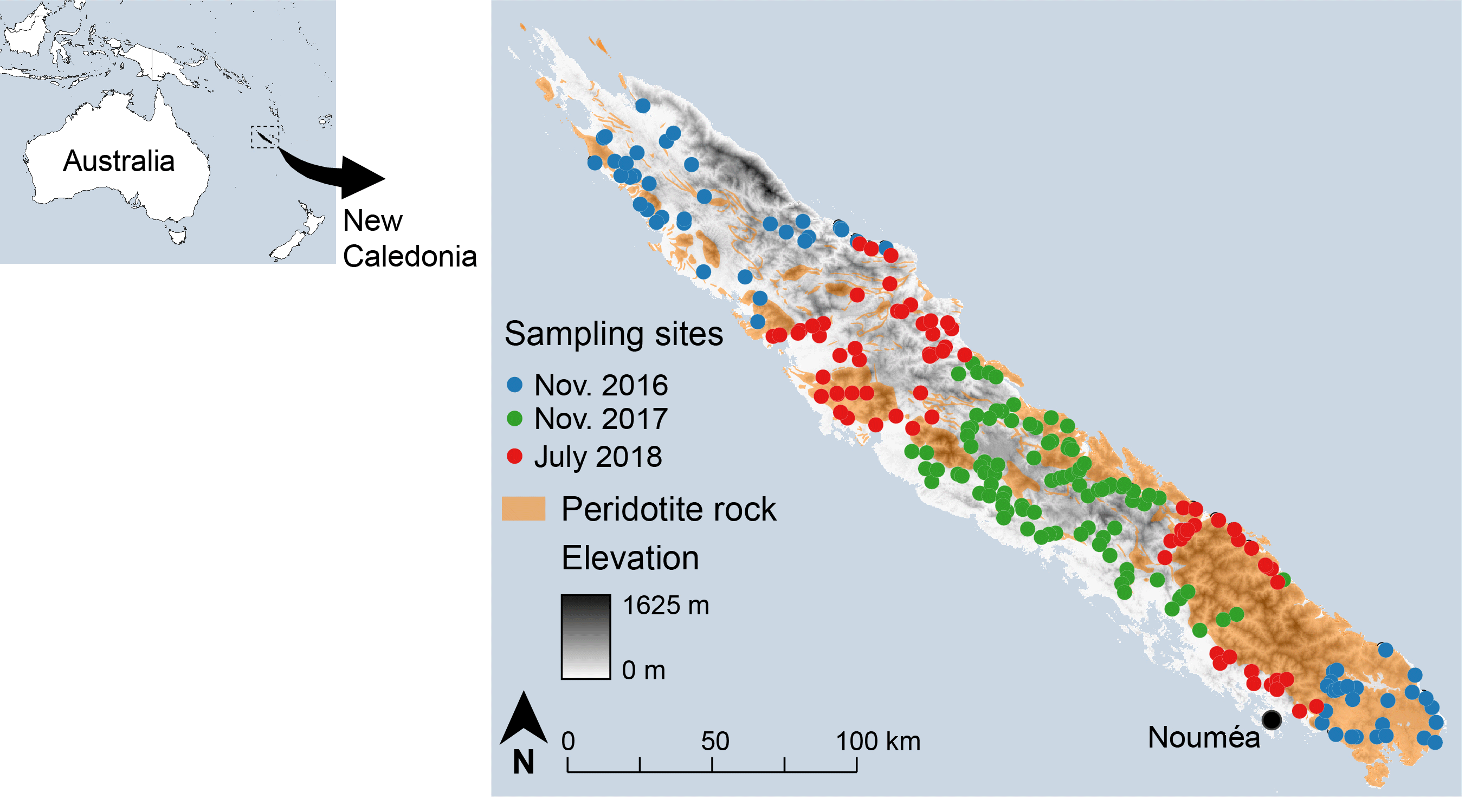


Local predictors

We measured nine local predictors to describe in-stream habitat conditions (Mouron et al., 2022): specific conductance, dissolved oxygen (DO), redox potential, pH, temperature of hyporheic water, stream width, mean annual air temperature, elevation and stream slope. We measured specific conductance, dissolved oxygen (DO), redox potential, pH, and temperature of hyporheic water with a multi-parameter probe (Odéon, Aqualabo, France). We measured stream width as the width of the area occupied by low-flow channels and unvegetated gravel bars (Bertrand & Liébault 2019). We extracted mean annual air temperature at the site from the WorldClim 2 dataset (30 arc-second resolution, Fick & Hijmans 2017). We obtained elevation of the sites from a 10-m resolution digital elevation model (DEM) of New Caledonia. We computed stream slope for every 50 m-long stream segment of the digital stream network of New Caledonia.

Catchment predictors

We used nine predictors to describe catchments (Mouron et al., 2022): the areal proportion of peridotite rocks, normalized difference vegetation index (NDVI), three land cover predictors (land cover predictors 1 to 3), areal proportion of surfaces eroded by mining activities, catchment area, mean annual precipitation, and low flow specific stream discharge.

We used the digital elevation model and digital stream network to delimit the upstream contributing catchment area associated with each sampling site (catchment area in km^2^). The network contained 29,405 nodes regularly located at every 0.1-km^2^ increase in catchment area from a minimum catchment area of 5 km^2^. We computed the areal proportion of peridotite rocks from the 1:50 000 geologic map of New Caledonia. We computed the mean NDVI for each catchment using 12 NDVI raster files available at a resolution of 250 m over the period 2000–2011.

We derived the three land cover predictors from the 1:12000 vector map of the land cover of New Caledonia. In each catchment, we quantified the areal proportions of six land cover classes: bare soil, herbaceous vegetation, shrubs, mature forests, urban area and water bodies. Then, we performed a correspondence analysis (CA) of the land cover composition for the 228 catchments and used the coordinates of catchments along the first three factorial axes of the analysis as three distinct land cover predictors (see Figure ST1.2 below). Positive coordinates along axes 1 and 2 were associated with a high proportion of bare soil whereas negative coordinates along axes 1 and 2 were associated with high proportion of mature forests and herbaceous vegetation, respectively. Coordinates along axis 3 increased with increasing proportion of shrubs. CA was performed in R with the ade4 package (Chessel et al., 2004).

We computed the areal proportion of surfaces eroded by mining activities for each catchment using a map of mining-degraded areas built from SPOT5 satellite images (Gouvernement de la Nouvelle-Calédonie, 2006). We extracted from the WorldClim 2 dataset (30 arc-second resolution, Fick & Hijmans, 2017) the mean annual precipitation averaged over the upstream contributing catchment area associated with each site. We obtained the low flow specific discharge for each catchment, defined as the average daily flow exceeded 355 days per year, from hydrological models by Romieux and Wotling (2016).

References

Bertrand, M., and F. Liébault. 2019. Active channel width as a proxy of sediment supply from mining sites in New Caledonia: Sediment supply from mining sites in New Caledonia. Earth Surface Processes and Landforms 44:67–76. <https://doi.org/10.1002/esp.4478>

Chessel, D., A.-B. Dufour, and J. Thioulouse. 2004. The ade4 Package – I: One-Table Methods. R News 4:5–10. <https://journal.r-project.org/articles/RN-2004-002/>

Fick, S. E., and R. J. Hijmans. 2017. WorldClim 2: new 1-km spatial resolution climate surfaces for global land areas. International Journal of Climatology 37:4302–4315. <https://doi.org/10.1002/joc.5086>

Gouvernement de la Nouvelle-Calédonie. 2006. Cartographie des surfaces dégradées par l'activité minière à l'aide de SPOT5. Direction de l’industrie, des mines et de l’Energie de la Nouvelle Calédonie (DIMENC) et Direction des Technologies et des Services de l'Information (DTSI). <https://dtsi-sgt.maps.arcgis.com/home/item.html?id=70a4476e211a4a6fa8c1772acd35c3db>

Mouron, S., D. Eme, A. Bellec, M. Bertrand, S. Mammola, F. Liébault, C. J. Douady, and F. Malard F. 2022. Unique and shared effects of local and catchment predictors over distribution of hyporheic organisms: does the valley rule the stream? Ecography, 5, e06099. <https://doi.org/10.1111/ecog.06099>

Romieux, N., and G. Wotling. 2016. Caractérisation des régimes d’étiage Actualisation des Débits Caractéristiques d’Etiages (DCE) Observations et Modélisations. DAVAR, Nouvelle-Calédonie.

Figure ST1.2: Positions of the 228 sampled sites (black dots) and six land cover classes along the first three axes of the correspondence analysis. Axes 1, 2 and 3 explained 49.9, 25.8 and 16.3 % of the variance, respectively.


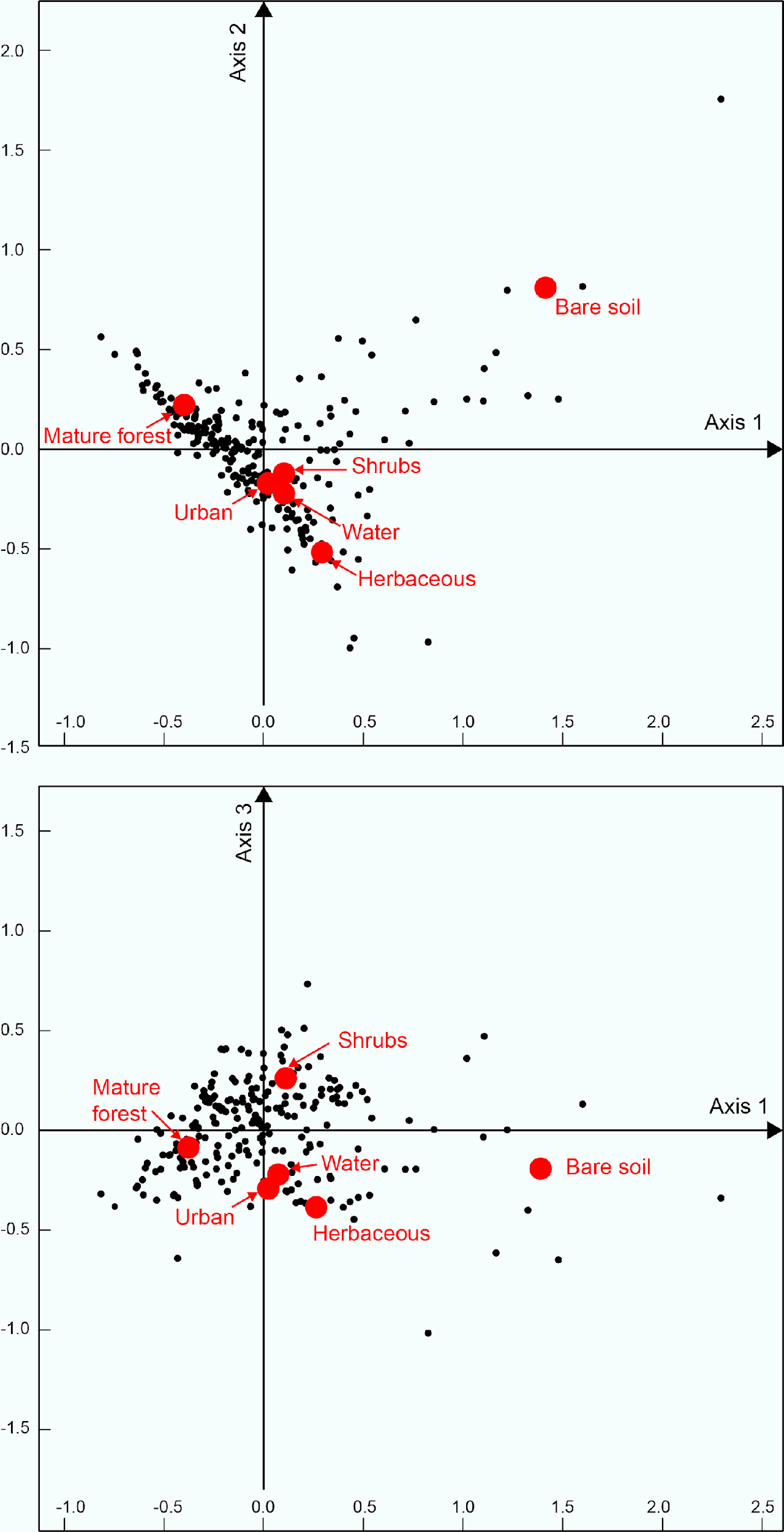


# Supplementary text 2: Model results when analyzing each year separately

We performed generalized linear models (GLM) on three data sets comprising the samples collected in 2016 (n=61 sites), 2017 (n=95 sites), and 2018 (n=72 sites). We found that peridotite in 2016 and 2018 and bare soils in 2018 were the first explanatory predictors in joint models for LSRt.

## Table ST2.1: Summary results of generalized linear models (GLM) for testing the effects of local and catchment predictors on local species richness (LSRt) using the three yearly data sets (2016: n=61 sites, 2017: n=95 sites, and 2018: n=72 sites). AICc: Akaike information criterion corrected for small sample size. Explained variation: proportion in reduction of deviance. In bold, significant p values for the predictors in the joint model. The superscript next to the predictor’s names indicates the quadratic form.

| Local species  Richness (LSRt) | Model | Predictors | AICc Weight | Explained variation (%) | Coefficient | p |
| --- | --- | --- | --- | --- | --- | --- |
| 2016 | Local | Intercept |  |  | 2.507 | < 0.001 |
|  |  | Stream width | 0.99 | 0.79 | 0.164 | 0.0084 |
|  |  | Dissolved oxygen | 0.72 | 2.95 | -0.333 | < 0.001 |
|  |  | Dissolved oxygen^2^ | 0.92 | 1.82 | -0.211 | 0.0088 |
|  |  | Elevation^2^ | 0.89 | 1.36 | -0.124 | 0.0281 |
|  | Catchment | Intercept |  |  | 2.185 | < 0.001 |
|  |  | Peridotite | 0.81 | 6.06 | -0.351 | < 0.001 |
|  | Joint | Intercept |  |  | 2.318 | < 0.001 |
|  |  | Peridotite | 1 | 6.06 | -0.425 | **< 0.001** |
|  |  | Stream width | 1 | 2.79 | 0.171 | **0.001** |
|  |  | Dissolved oxygen | 0.36 | 0.06 | 0.059 | 0.6432 |
|  |  | Dissolved oxygen^2^ | 0.52 | 0.93 | -0.091 | 0.2427 |
|  |  | Elevation^2^ | 0.46 | 0.68 | -0.076 | 0.1361 |
| 2017 | Local | Intercept |  |  | 2.152 | < 0.001 |
|  |  | pH | 1 | 1.52 | -0.194 | 0.0027 |
|  | Catchment | Intercept |  |  | 2.07 | < 0.001 |
|  |  | Land cover 1 | 0.65 | 1 | -0.316 | < 0.001 |
|  |  | Land cover 1^2^ | 0.64 | 1.62 | 0.071 | 0.0028 |
|  | Joint | Intercept |  |  | 2.078 | < 0.001 |
|  |  | Land cover 1 | 0.94 | 0.45 | -0.257 | **0.0036** |
|  |  | Land cover 1^2^ | 0.87 | 1.03 | 0.06 | **0.0173** |
|  |  | pH | 0.55 | 1.52 | -0.101 | 0.1451 |
| 2018 | Local | Intercept |  |  | 2.283 | < 0.001 |
|  |  | Stream slope | 0.76 | 1.14 | -0.137 | 0.0273 |
|  | Catchment | Intercept |  |  | 2.516 | < 0.001 |
|  |  | Peridotite | 1 | 4.35 | -0.247 | < 0.001 |
|  |  | Peridotite^2^ | 0.95 | 1.22 | -0.227 | 0.0083 |
|  |  | Catchment area^2^ | 0.92 | 0.91 | -0.04 | 0.0589 |
|  | Joint | Intercept |  |  | 2.502 | < 0.001 |
|  |  | Peridotite | 1 | 3.62 | -0.232 | **< 0.001** |
|  |  | Peridotite^2^ | 0.84 | 1.07 | -0.214 | **0.0128** |
|  |  | Catchment area^2^ | 0.64 | 0.98 | -0.041 | 0.0503 |
|  |  | Stream slope | 0.36 | 1.14 | -0.062 | 0.2733 |

# Supplementary text 3: Model results for the subset of sites with areal proportion of peridotite rocks ≥ 85 %

Nickel mining in New Caledonia occurs exclusively in peridotite catchments. Hence, to tease apart the potential effect of the areal proportion of surfaces eroded by mining activities on LSR from that of the areal proportion of peridotite rocks, we performed generalized linear models (GLMs) on a subset of sites whose catchments had an areal proportion of peridotite rocks ≥ 85 % (n=63 sites). We provide below the results of the different steps of the analysis, including:

- Table ST3.1: selection of predictors using generalized additive models (GAMs)

- Table ST3.2: selection of GLMs distributions for modelling local species richness as a function of local and catchment predictors, which were selected using GAMs.

- Table ST3.3: results of generalized linear models for testing the effects of local and catchment predictors on local species richness

- Figure ST3.4. Significant relationships between local species richness (LSR) and predictors

## Table ST3.1: Results of generalized additive models (GAMs) performed to select predictors to include in subsequent GLMs of local species richness (n=63 sites with an areal proportion of peridotite rocks in the catchment ≥ 85 %). Only significant predictors (shown in bold) and the areal proportion of mining-degraded areas were included in subsequent GLMs. EDF: effective degree of freedom; Explained variation: proportion in reduction of deviance.

| Local species  Richness (LSR) | Model | Predictors | EDF | p value | Explained variation |
| --- | --- | --- | --- | --- | --- |
| Community (LSRt) | Local | **Redox potential** | **1.103** | **0.011** | **5.531** |
|  |  | Stream slope | 1.521 | 0.0942 | 3.273 |
|  |  | pH | 1.187 | 0.0742 | 2.991 |
|  |  | Dissolved oxygen (DO) | 1.522 | 0.3252 | 2.142 |
|  |  | Specific conductance | 1 | 0.1386 | 1.668 |
|  |  | Temperature | 1.203 | 0.4971 | 0.861 |
|  |  | Stream width | 1 | 0.2686 | 0.844 |
|  |  | Mean annual air temperature | 1 | 0.2951 | 0.775 |
|  |  | Elevation | 1.001 | 0.8261 | 0.037 |
|  | Catchment | **Precipitation** | **1.838** | **0.0375** | **5.101** |
|  |  | Land cover1 | 1.813 | 0.111 | 3.633 |
|  |  | **Proportion of mining-degraded areas** | **1** | **0.0423** | **3.299** |
|  |  | Normalized difference vegetation index | 1.496 | 0.1547 | 3.023 |
|  |  | Land cover 3 | 1 | 0.092 | 1.982 |
|  |  | Land cover 2 | 1.369 | 0.4441 | 1.312 |
|  |  | Discharge | 1 | 0.3749 | 0.555 |
|  |  | Areal proportion of peridotite | 1 | 0.6775 | 0.124 |
|  |  | Catchment_area | 1 | 0.8981 | 0.012 |
| Fast-growing species (LSRf) | Local | **Temperature** | **1.843** | **0.0716** | **5.561** |
|  |  | Mean annual air temperature | 1.521 | 0.0806 | 5.056 |
|  |  | Dissolved oxygen (DO) | 1.466 | 0.1147 | 4.723 |
|  |  | Elevation | 1.743 | 0.2009 | 3.471 |
|  |  | Stream slope | 1 | 0.0294 | 3.334 |
|  |  | Redox potential | 1 | 0.0803 | 2.778 |
|  |  | Specific conductance | 1 | 0.2531 | 1.204 |
|  |  | pH | 1 | 0.485 | 0.415 |
|  |  | Stream width | 1.039 | 0.5663 | 0.404 |
|  | Catchment | **Precipitation** | **1.815** | **0.0456** | **5.735** |
|  |  | Areal proportion of peridotite | 1.767 | 0.064 | 5.088 |
|  |  | Land cover 2 | 1.729 | 0.2421 | 2.994 |
|  |  | Discharge | 1.385 | 0.3466 | 2.046 |
|  |  | **Proportion of mining-degraded areas** | **1** | **0.1938** | **1.604** |
|  |  | Land cover 3 | 1 | 0.173 | 1.56 |
|  |  | Land cover 1 | 1 | 0.1736 | 1.525 |
|  |  | Catchment area | 1 | 0.5065 | 0.403 |
|  |  | Normalized difference vegetation index | 1.001 | 0.6311 | 0.197 |
| Slow-growing species (LSRs) | Local | Specific conductance | 1.815 | 0.0853 | 7.871 |
|  |  | **Stream width** | **1** | **0.033** | **5.536** |
|  |  | Redox potential | 1.518 | 0.1611 | 5.529 |
|  |  | Temperature | 1 | 0.1253 | 3.103 |
|  |  | pH | 1 | 0.1841 | 2.277 |
|  |  | Dissolved oxygen (DO) | 1 | 0.4464 | 0.734 |
|  |  | Mean annual air temperature | 1 | 0.4534 | 0.733 |
|  |  | Stream slope | 1 | 0.5072 | 0.637 |
|  |  | Elevation | 1 | 0.5692 | 0.435 |
|  | Catchment | **Normalized difference vegetation index** | **1** | **0.0022** | **13.334** |
|  |  | **Precipitation** | **1** | **0.0045** | **10.323** |
|  |  | **Land cover 1** | **1** | **0.0206** | **7.626** |
|  |  | Areal proportion of peridotite | 1.378 | 0.2716 | 3.873 |
|  |  | **Proportion of mining-degraded areas** | **1** | **0.2498** | **1.917** |
|  |  | Discharge | 1 | 0.3751 | 1.018 |
|  |  | Catchment area | 1 | 0.3759 | 0.949 |
|  |  | Land cover 2 | 1 | 0.7548 | 0.129 |
|  |  | Land cover 3 | 1 | 0.9298 | 0.01 |

## Table ST3.2: Selection of GLMs distributions for modelling local species richness as a function of local and catchment predictors for the subset of sites with an areal proportion of peridotite rocks in the catchment ≥ 85 % (n=63 sites). AICc: Akaike information criterion corrected for small sample size. Selected distributions are shown in bold.

| Local species  Richness (LSR) | Distribution | AICc | Delta AICc |
| --- | --- | --- | --- |
| Community (LSRt) | Poisson | 349.3 |  |
|  | Zero-Inflated Poisson | 344.5 | -4.8 |
|  | Zero-Inflated Negative Binomial | 334.8 | -9.7 |
|  | **Negative Binomial** | **333.7** | **-1.1** |
| Fast growing species (LSRf) | Zero-Inflated Poisson | 285.2 |  |
|  | Poisson | 282.8 | -2.3 |
|  | Zero-Inflated Negative Binomial | 279.2 | -3.6 |
|  | **Negative Binomial** | **276.6** | **-2.6** |
| Slow growing species (LSRs) | Zero-Inflated Negative Binomial | 239.4 |  |
|  | Zero-Inflated Poisson | 236.8 | -2.6 |
|  | Negative Binomial | 236.8 | 0 |
|  | **Poisson** | **234.2** | **-2.5** |

## Table ST3.3: Summary results of generalized linear models (GLM) for testing the effects of local and catchment predictors on local species richness (LSR) using a subset of sites with an areal proportion of peridotite rocks in the catchment ≥ 85 % (n=63 sites). AICc: Akaike information criterion corrected for small sample size. Explained variation: proportion in reduction of deviance. In bold, significant p values for the predictors. NDVI: normalized difference vegetation index. The superscript next to the predictor’s names indicates the quadratic form. The variation in LSR accounted for by the GLM for the total community, fast-growing species and slow growing species is expressed using the Nagelkerke’s pseudo R^2^ (R²_N_).

| Local species  Richness (LSR) | Predictors | AICc  weight | Explained  variation (%) | Coefficient | p |
| --- | --- | --- | --- | --- | --- |
| Community (LSRt) | Intercept |  |  | 1.716 | < 0.001 |
| (R²_N_ = 0.11) | Redox potential | 0.55 | 1.03 | 0.111 | 0.1518 |
|  | Precipitation^2^ | 0.53 | 0.68 | 0.072 | 0.1165 |
|  | Proportion of mining-degraded areas | 0.45 | 0.52 | -0.112 | 0.1959 |
|  | Precipitation | 0.25 | 0.01 | -0.016 | 0.8330 |
| Fast-growing species (LSRf) | Intercept |  |  | 1.157 | < 0.001 |
| (R²_N_ = 0.14) | Temperature^2^ | 0.54 | 1.06 | -0.134 | 0.1397 |
|  | Precipitation^2^ | 0.51 | 0.7 | 0.097 | 0.0885 |
|  | Proportion of mining-degraded areas | 0.45 | 0.62 | -0.207 | 0.0653 |
|  | Precipitation | 0.39 | 0.88 | -0.162 | 0.1011 |
|  | Temperature | 0.26 | 0.19 | 0.071 | 0.4859 |
| Slow-growing species (LSRs) | Intercept |  |  | 0.923 | < 0.001 |
| (R²_N_ = 0.25) | NDVI | 0.84 | 4.27 | 0.485 | **0.0174** |
|  | Stream width | 0.63 | 1.21 | 0.152 | **0.0388** |
|  | Precipitation | 0.53 | 0.71 | 0.165 | 0.0887 |
|  | Landcover1 | 0.45 | 1.40 | 0.360 | 0.0791 |
|  | Proportion of mining-degraded areas | 0.23 | 0.02 | 0.020 | 0.8538 |

## Figure ST3.4: Significant relationships (p < 0.05) between the local species richness (LSR) of slow-growing species (LSRs) and the normalized difference vegetation index (NDVI) and stream width for the subset of sites with an areal proportion of sites ≥ 85 % (n=63 sites). Equations are from generalized models with a Poisson distribution, performed using normalized values of predictors.


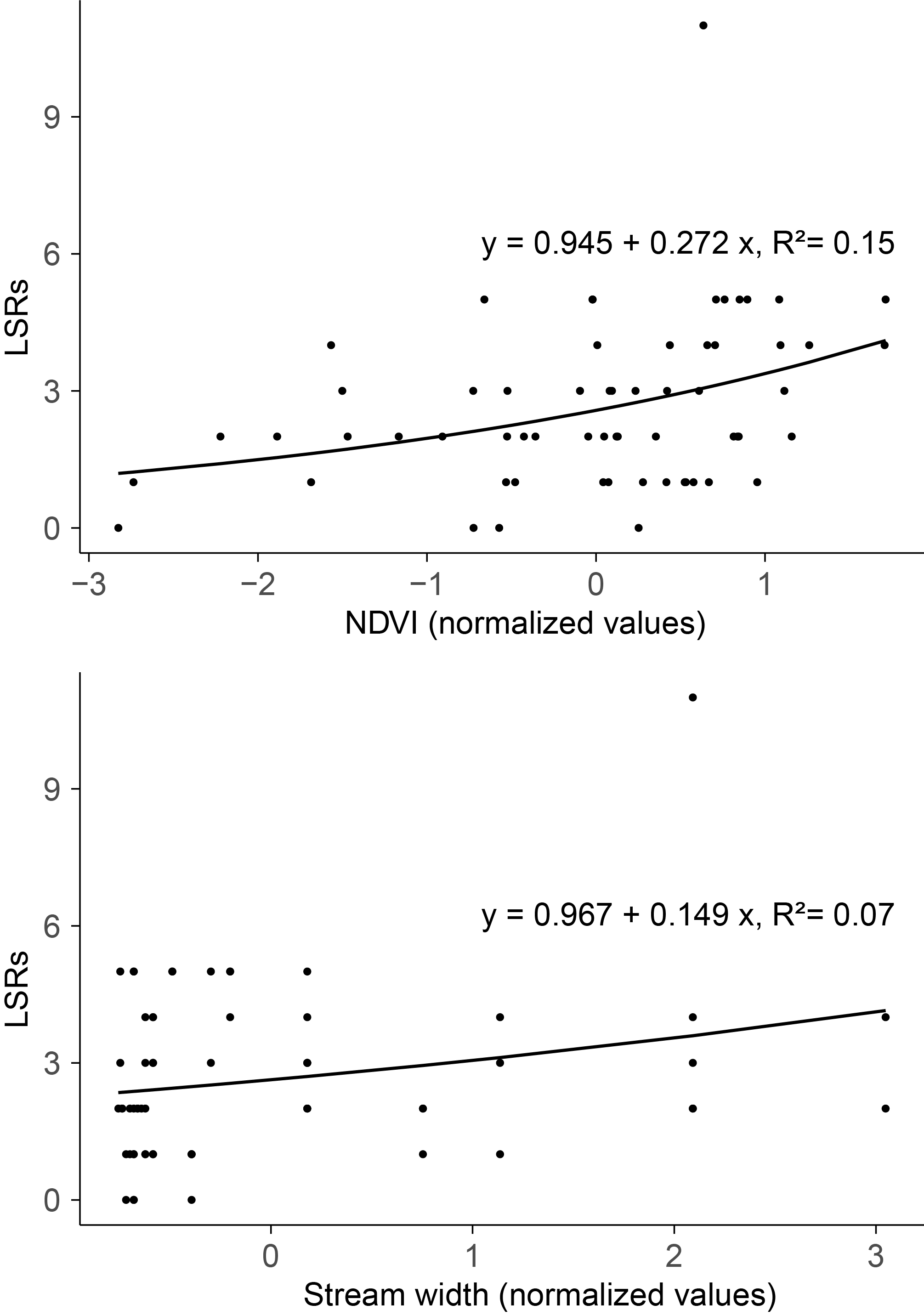


# Table S1: Sample – species data

Available at: <https://zenodo.org/records/17083758>

# Table S2: Environmental predictors and response variables

Available at: <https://zenodo.org/records/17083758>

# Table S3: Pearson's correlation coefficients among predictors.

Land cover 1, 2 and 3 correspond to the coordinates of catchments along the first three factorial axes of the land cover correspondence analysis (see supplementary text S1). Significant correlations (p<0.05) are shown in bold.


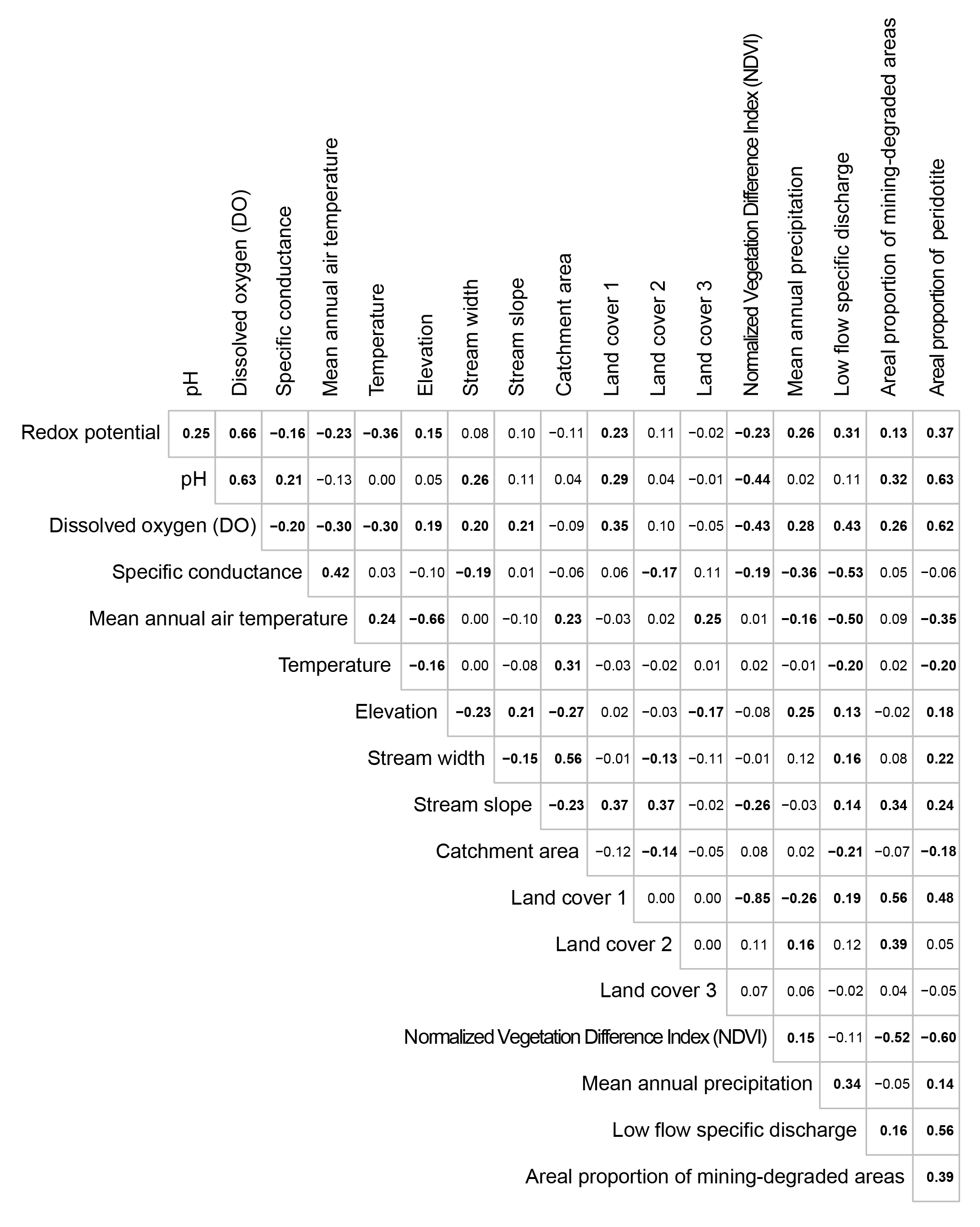


# Table S4: Variance inflation factors

Variance inflation factors (VIF) between any predictor and all other local (VIFlocal), catchment (VIFcatchment) and local and catchment predictors (VIFlocal & catchment). Values above 5 are indicative of multicollinearity. NA: not applicable

| Predictors | Predictor type | VIF local | VIF catchment | VIF local & catchment |
| --- | --- | --- | --- | --- |
| Stream width | Local | 1.29 | NA | 2.21 |
| Elevation | Local | 2.09 | NA | 2.77 |
| Specific conductance | Local | 1.92 | NA | 2.42 |
| Redox potential | Local | 2.01 | NA | 2.11 |
| pH | Local | 2.76 | NA | 3.79 |
| Dissolved oxygen (DO) | Local | 3.93 | NA | 4.54 |
| Temperature | Local | 1.32 | NA | 1.52 |
| Mean annual air temperature | Local | 2.56 | NA | 3.72 |
| Stream slope | Local | 1.12 | NA | 1.56 |
| Areal proportion of peridotite | Catchment | NA | 2.75 | 4.65 |
| Catchment area | Catchment | NA | 1.10 | 2.05 |
| Land cover 1 | Catchment | NA | 5.06 | 5.80 |
| Land cover 2 | Catchment | NA | 1.52 | 1.87 |
| Land cover 3 | Catchment | NA | 1.05 | 1.20 |
| Normalized vegetation difference index (NDVI) | Catchment | NA | 6.17 | 6.99 |
| Low flow specific discharge | Catchment | NA | 2.09 | 3.37 |
| Precipitation | Catchment | NA | 1.42 | 1.92 |
| Areal proportion of mining-degraded areas | Catchment | NA | 2.02 | 2.22 |

# Table S5: Selection of model distributions

Selection of GLMs distributions for modelling local species richness as a function of local and catchment predictors. AICc: Akaike information criterion corrected for small sample size. Selected distributions are shown in bold.

| Local species  Richness (LSR) | Model | Distribution | AICc | Delta  AICc |
| --- | --- | --- | --- | --- |
| Community (LSRt) | Local | Poisson | 1513.2 |  |
|  |  | Zero-Inflated Poisson | 1505 | -8.2 |
|  |  | Zero-Inflated Negative Binomial | 1370.8 | -134.3 |
|  |  | **Negative Binomial** | **1368.7** | **-2** |
|  | Catchment | Poisson | 1457.7 |  |
|  |  | Zero-Inflated Poisson | 1445.9 | -11.7 |
|  |  | Zero-Inflated Negative Binomial | 1349.4 | -96.5 |
|  |  | **Negative Binomial** | **1348.7** | **-0.7** |
| Fast growing species (LSRf) | Local | Poisson | 1563.5 |  |
|  |  | Zero-Inflated Poisson | 1553.6 | -9.9 |
|  |  | Zero-Inflated Negative Binomial | 1337.6 | -215.9 |
|  |  | **Negative Binomial** | **1335.3** | **-2.3** |
|  | Catchment | Poisson | 1426.8 |  |
|  |  | Zero-Inflated Poisson | 1413.4 | -13.4 |
|  |  | Zero-Inflated Negative Binomial | 1287.5 | -125.9 |
|  |  | **Negative Binomial** | **1285.3** | **-2.2** |
| Slow growing species (LSRs) | Local | Zero-Inflated Negative Binomial | 808.6 |  |
|  |  | Negative Binomial | 806.3 | -2.3 |
|  |  | Zero-Inflated Poisson | 806.3 | 0 |
|  |  | **Poisson** | **804** | **-2.3** |
|  | Catchment | Zero-Inflated Negative Binomial | 833.2 |  |
|  |  | Zero-Inflated Poisson | 831 | -2.2 |
|  |  | Negative Binomial | 831 | -0.1 |
|  |  | **Poisson** | **828.8** | **-2.2** |

# **Table S6:** Results of generalized additive models (GAMs) to assess nonlinearity.

Predictors with an effective degree of freedom (EDF) higher than 1.5 and p value ≤ 0.05 (shown in bold) were included in both their linear and quadratic forms in subsequent GLMs of local species richness. Ref.df: reference degrees of freedom. Chi.sq: Chi-Square Statistic.

| Local species  richness (LSR) | Predictor  type | Predictor | EDF | Ref.df | Chi.sq | p-value |
| --- | --- | --- | --- | --- | --- | --- |
| Community (LSRt) | Local | Stream width | 1.000 | 1.000 | 1.120 | 0.2899 |
|  |  | Elevation | 1.893 | 1.988 | 10.396 | **0.0045** |
|  |  | Specific conductance | 1.277 | 1.477 | 1.057 | 0.5463 |
|  |  | Redox potential | 1.000 | 1.000 | 2.830 | 0.0925 |
|  |  | pH | 1.000 | 1.000 | 17.278 | < 0.001 |
|  |  | Dissolved oxygen (DO) | 1.909 | 1.991 | 10.811 | **0.0053** |
|  |  | Temperature | 1.892 | 1.988 | 8.888 | **0.0103** |
|  |  | Mean annual air temperature | 1.000 | 1.000 | 0.197 | 0.6579 |
|  |  | Stream slope | 1.797 | 1.959 | 4.492 | 0.1102 |
|  | Catchment | Areal proportion of peridotite | 1.914 | 1.992 | 46.965 | **< 0.001** |
|  |  | Catchment area | 1.920 | 1.994 | 12.306 | **0.0030** |
|  |  | Land cover 1 | 1.999 | 2.000 | 16.778 | **< 0.001** |
|  |  | Land cover 2 | 1.140 | 1.260 | 0.056 | 0.9450 |
|  |  | Land cover 3 | 1.000 | 1.000 | 0.173 | 0.6771 |
|  |  | NDVI | 1.756 | 1.940 | 10.401 | **0.0105** |
|  |  | Discharge | 1.000 | 1.000 | 2.635 | 0.1045 |
|  |  | Precipitation | 1.004 | 1.008 | 3.452 | 0.0633 |
|  |  | Areal proportion of mining-degraded areas | 1.001 | 1.002 | 0.261 | 0.6093 |
| Fast-growing species (LSRf) | Local | Stream width | 1.539 | 1.787 | 10.125 | **0.0030** |
|  |  | Elevation | 1.897 | 1.989 | 9.544 | **0.0073** |
|  |  | Specific conductance | 1.830 | 1.971 | 10.632 | **0.0061** |
|  |  | Redox potential | 1.000 | 1.000 | 5.709 | 0.0169 |
|  |  | pH | 1.000 | 1.000 | 8.910 | 0.0028 |
|  |  | Dissolved oxygen (DO) | 1.970 | 1.999 | 29.216 | **< 0.001** |
|  |  | Temperature | 1.800 | 1.959 | 4.307 | 0.0994 |
|  |  | Mean annual air temperature | 1.001 | 1.001 | 4.540 | 0.0332 |
|  |  | Stream slope | 1.832 | 1.971 | 8.768 | **0.0215** |
|  | Catchment | Areal proportion of peridotite | 1.944 | 1.997 | 123.595 | **< 0.001** |
|  |  | Catchment area | 1.964 | 1.999 | 31.521 | **< 0.001** |
|  |  | Land cover 1 | 1.992 | 1.999 | 29.121 | **< 0.001** |
|  |  | Land cover 2 | 1.076 | 1.146 | 0.030 | 0.9690 |
|  |  | Land cover 3 | 1.000 | 1.000 | 0.211 | 0.6464 |
|  |  | NDVI | 1.213 | 1.379 | 22.143 | < 0.001 |
|  |  | Discharge | 1.000 | 1.000 | 13.049 | < 0.001 |
|  |  | Precipitation | 1.000 | 1.000 | 0.023 | 0.8795 |
|  |  | Areal proportion of mining-degraded areas | 1.592 | 1.833 | 1.462 | 0.5023 |
| Slow-growing species (LSRs) | Local | Stream width | 1.000 | 1.000 | 9.922 | 0.0016 |
|  |  | Elevation | 1.830 | 1.971 | 6.222 | **0.0315** |
|  |  | Specific conductance | 1.000 | 1.000 | 4.277 | 0.0386 |
|  |  | Redox potential | 1.522 | 1.770 | 9.690 | **0.0036** |
|  |  | pH | 1.764 | 1.940 | 4.361 | 0.1472 |
|  |  | Dissolved oxygen (DO) | 1.435 | 1.675 | 0.775 | 0.6420 |
|  |  | Temperature | 1.000 | 1.000 | 0.007 | 0.9313 |
|  |  | Mean annual air temperature | 1.999 | 2.000 | 11.519 | **0.0032** |
|  |  | Stream slope | 1.000 | 1.000 | 1.775 | 0.1827 |
|  | Catchment | Areal proportion of peridotite | 1.906 | 1.991 | 16.126 | **< 0.001** |
|  |  | Catchment area | 1.000 | 1.000 | 0.598 | 0.4395 |
|  |  | Land cover 1 | 1.000 | 1.000 | 0.081 | 0.7755 |
|  |  | Land cover 2 | 1.000 | 1.000 | 0.286 | 0.5930 |
|  |  | Land cover 3 | 1.000 | 1.000 | 0.001 | 0.9751 |
|  |  | NDVI | 1.000 | 1.000 | 0.027 | 0.8707 |
|  |  | Discharge | 1.852 | 1.978 | 5.995 | 0.0637 |
|  |  | Precipitation | 1.000 | 1.000 | 4.332 | 0.0374 |
|  |  | Areal proportion of mining-degraded areas | 1.000 | 1.000 | 0.106 | 0.7444 |

# Table S7: Model results for the relationships among LSR, abundance and catchment productivity

Results of linear models for the relationships among LSR, abundance and catchment productivity. LSRt, LSRf, and LSRs are local species richness for the total community, fast-growing species, and slow-growing species, respectively. The same applies to abundance-t, -f, and –s. Peridotite: areal proportion of peridotite rocks. NDVI: normalized difference vegetation index. The superscript next to peridotite and NDVI indicates the quadratic form. LR Chisq: value of the Chi-Square Statistic for the likelihood ratio test used to test for the inclusion of a quadratic term in the models. LR p value: p value of the likelihood ratio test.

| Response variable | Term | Estimate | p value | Model  R^2^ | Model  p value | LR  Chisq | LR  p value |
| --- | --- | --- | --- | --- | --- | --- | --- |
| LSRt | Intercept | 2.430 | < 0.001 | 0.15 | < 0.001 | 0.858 | 0.3544 |
| LSRt | Peridotite | -0.006 | < 0.001 |  |  |  |  |
| LSRt | Intercept | 0.190 | 0.6525 | 0.09 | < 0.001 | 2.373 | 0.1234 |
| LSRt | NDVI | 2.649 | < 0.001 |  |  |  |  |
| LSRf | Intercept | 2.338 | < 0.001 | 0.30 | < 0.001 | 1.748 | 0.1861 |
| LSRf | Peridotite | -0.010 | < 0.001 |  |  |  |  |
| LSRf | Intercept | 8.671 | 0.0188 | 0.15 | < 0.001 | 7.204 | 0.0073 |
| LSRf | NDVI | -23.334 | 0.0228 |  |  |  |  |
| LSRf | NDVI^2^ | 18.909 | 0.0077 |  |  |  |  |
| LSRs | Intercept | 0.833 | < 0.001 | 0.09 | < 0.001 | 7.783 | 0.0053 |
| LSRs | Peridotite | 0.014 | < 0.001 |  |  |  |  |
| LSRs | Peridotite^2^ | -1.1e-04 | 0.0056 |  |  |  |  |
| LSRs | Intercept | -4.128 | 0.1376 | 0.04 | 0.0131 | 4.769 | 0.0290 |
| LSRs | NDVI | 15.707 | 0.0425 |  |  |  |  |
| LSRs | NDVI^2^ | -11.607 | 0.0302 |  |  |  |  |
| Abundance-t | Intercept | 4.333 | < 0.001 | 0.13 | < 0.001 | 3.114 | 0.0776 |
| Abundance-t | Peridotite | -0.010 | < 0.001 |  |  |  |  |
| Abundance-t | Intercept | 0.324 | 0.6760 | 0.09 | < 0.001 | 0 | 0.9995 |
| Abundance-t | NDVI | 4.781 | < 0.001 |  |  |  |  |
| Abundance-f | Intercept | 3.906 | < 0.001 | 0.35 | < 0.001 | 0.386 | 0.5341 |
| Abundance-f | Peridotite | -0.018 | < 0.001 |  |  |  |  |
| Abundance-f | Intercept | -2.759 | 0.0014 | 0.17 | < 0.001 | 3.715 | 0.0539 |
| Abundance-f | NDVI | 7.805 | < 0.001 |  |  |  |  |
| Abundance-s | Intercept | 2.224 | < 0.001 | 0.06 | < 0.001 | 11.623 | < 0.001 |
| Abundance-s | Peridotite | 0.044 | < 0.001 |  |  |  |  |
| Abundance-s | Peridotite^2^ | -4.0e-04 | < 0.001 |  |  |  |  |
| Abundance-s | Intercept | 3.299 | 0.0039 | < 0.01 | 0.6197 | 2.102 | 0.1471 |
| Abundance-s | NDVI | -0.745 | 0.6197 |  |  |  |  |
| LSRt | Intercept | 1.010 | < 0.001 | 0.30 | < 0.001 |  |  |
| LSRt | Abundance-t | 0.299 | < 0.001 |  |  |  |  |
| LSRf | Intercept | 0.549 | < 0.001 | 0.53 | < 0.001 |  |  |
| LSRf | Abundance-f | 0.434 | < 0.001 |  |  |  |  |
| LSRs | Intercept | 0.381 | < 0.001 | 0.52 | < 0.001 |  |  |
| LSRs | Abundance-s | 0.251 | < 0.001 |  |  |  |  |

# Figure S1: AICc weights of predictors

AICc weights of local (upper panels) and catchment (lower panels) predictors for the local species richness of the total community (LSRt, left panels), fast-growing species (LSRf, middle panels), and slow-growing species (LSRs, right panels), as obtained using multi-model inferences. Local and catchment models were built using predictors with an AICc weight > 0.7 (red vertical line). DO: dissolved oxygen; Mean ann. temp.: mean annual air temperature; Sp. conductance: specific conductance; Peridotite: areal proportion of peridotite rocks; NDVI: normalized difference vegetation index; Mine areas: areal proportions of mining-degraded areas. The superscript next to the predictor’s names indicates the quadratic form.


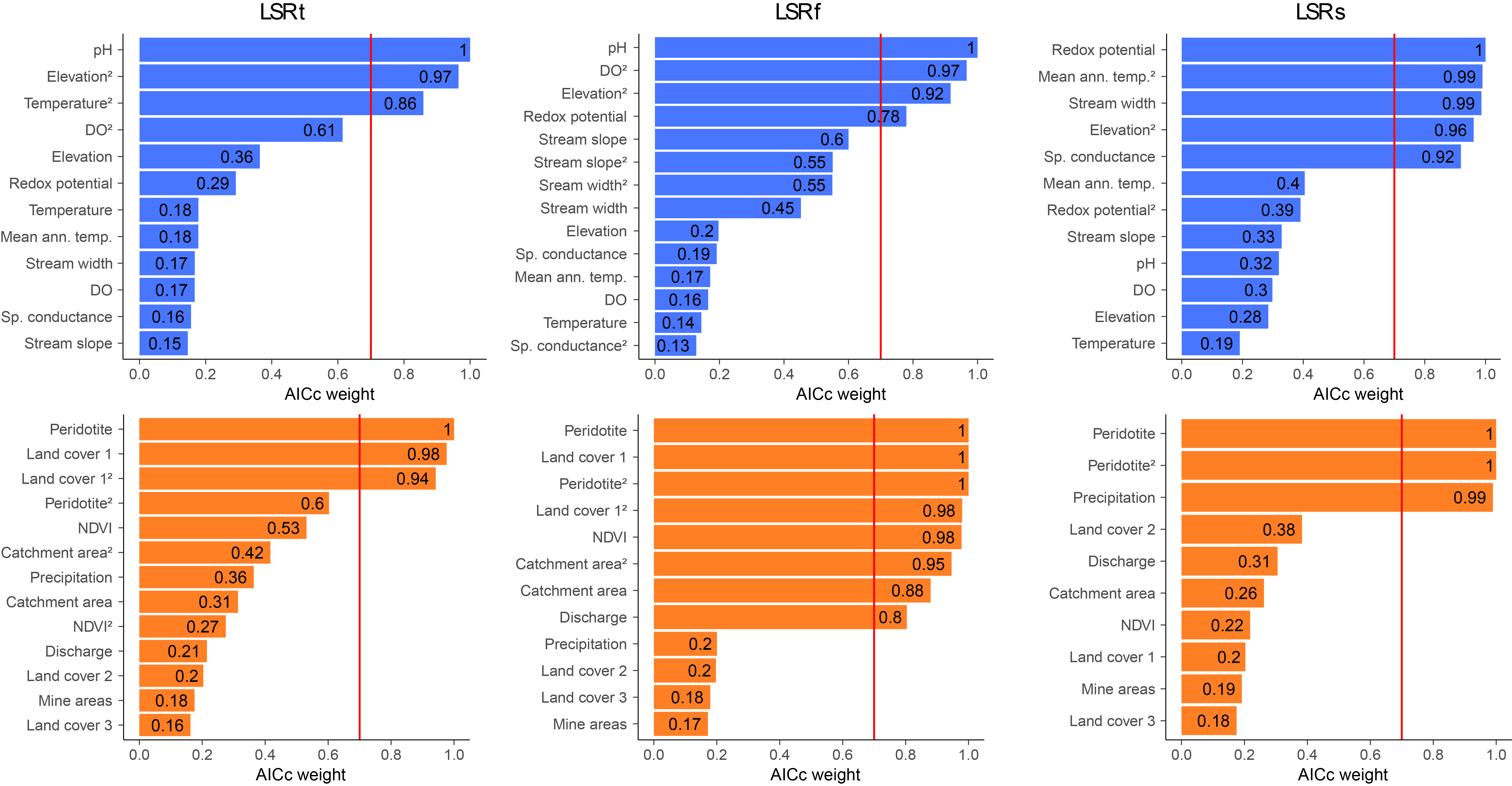


# Figure S2: Relationships between local species richness (LSR) and local and catchment predictors.

LSRt, LSRf, and LSRs are local species richness for the total community, fast-growing species, and slow-growing species, respectively. Equations are from generalized linear models with a negative binomial distribution for LSRt and LSRf and a Poisson distribution for LSRs, performed using normalized values of predictors. All relationships are significant (p < 0.05).


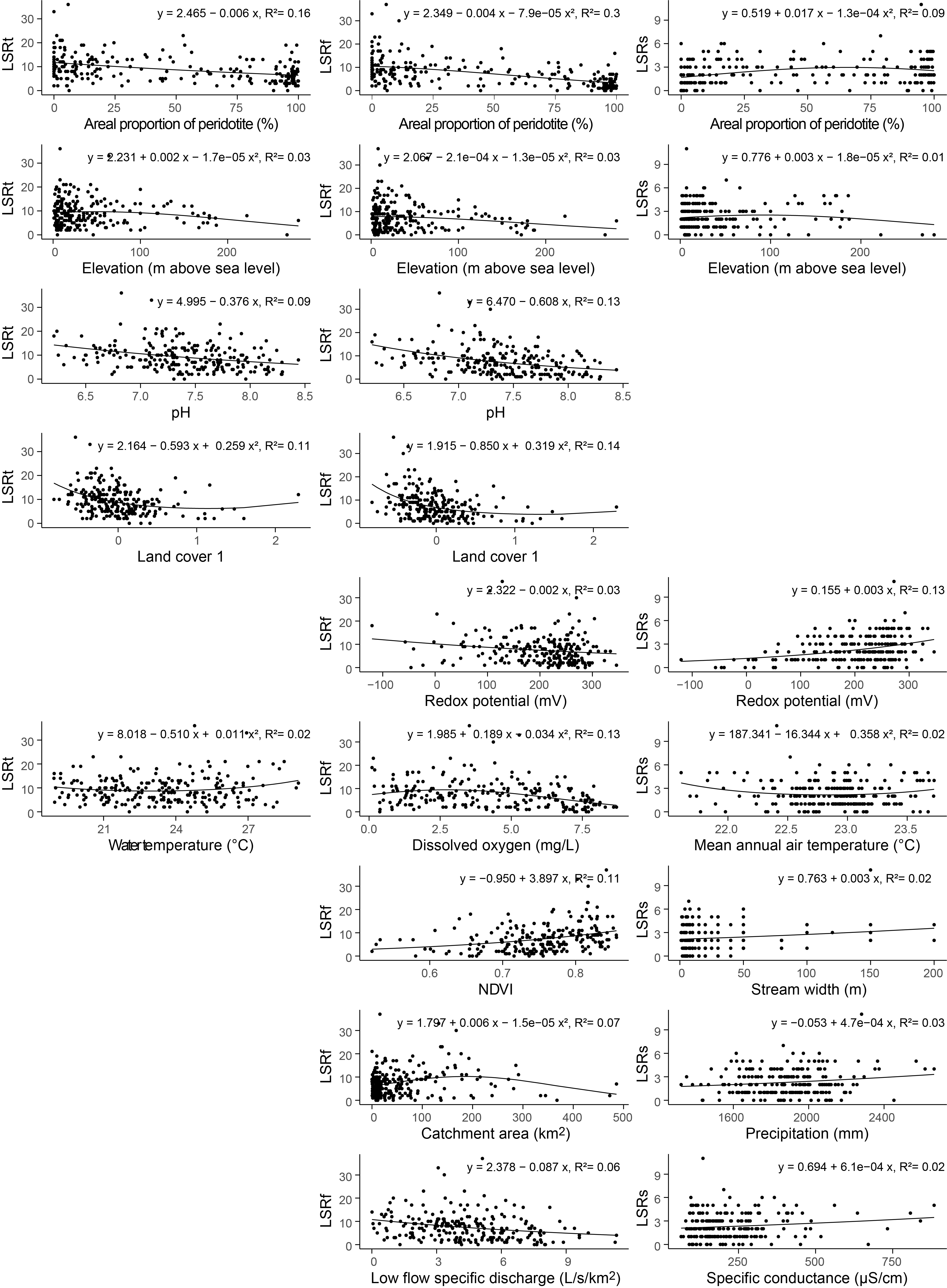


# Codes for analyses

R codes for replicating the analyses are available at: <https://zenodo.org/records/17083758>
